# Supplementary material for: The role of citizen science in mosquito-borne disease surveillance and control: A scoping review
Source: PLoS One. 2026 May 15;21(5):e0348697. doi: 10.1371/journal.pone.0348697 (PMC13178890; doi:10.1371/journal.pone.0348697)
Supplement: S2 File — (DOCX) [file pone.0348697.s002.docx]

**Supplementary file 2.** Detailed extracted information of the included articles

|  | Study Characteristics | | | | | | | Citizen-Science Framework | | | | | | | Data and tools/technologies | | | | | | | limitations and Ethics | | | | |
| --- | --- | --- | --- | --- | --- | --- | --- | --- | --- | --- | --- | --- | --- | --- | --- | --- | --- | --- | --- | --- | --- | --- | --- | --- | --- | --- |
| No. | Title | First Author | Year of Publication | Country | State | Study Setting | Study type | Participant Sample size | Age | Gender | race/ethnicity | Level of citizen involvement | Recruitment method | Purpose of Citizen Participation | disease | Tools, Platforms, and Technologies | Type of data | Validation mechanism | Statistical analysis | Effort-bias handling | Predictive or epidemiological outputs | limitations | Reported Biases | Study-reported ethics | Platform-level ethics | Link to dataset |
| [1](https://doi.org/10.1186/s13071-025-06774-3) | Spatial inference of Culex pipiens abundance and biting activity distribution in the Netherlands using citizen science | Ayat Abourashed | 2025 | Netherlands | N/A | Urban/rural | Descriptive ecological modelling study | General public using Mosquito Alert app; 14,405 Culex reports and 6,941 bite reports | N/A | N/A | N/A | Data collection | Open call; via national media (NOS) | Mosquito surveillance; biting activity monitoring | Culex pipiens (vectors of West Nile virus (WNV) and Usutu virus (USUV)) | Mosquito Alert mobile app; smartphone photos; expert validation; R software | Image reports; bite reports; geo-referenced data; environmental data | Distributed expert / specialist network validation; app-submitted mosquito images; three independent expert entomologists manually reviewed photographs, with senior entomologist review when needed; bite reports not validated because they lacked images | Generalized linear mixed models; AIC/BIC; VIF; cross-validation; pseudo-R2 | Explicit analytical adjustment for effort/bias; sampling effort was estimated per cell from participant reporting probability and incorporated into GLMMs as an offset to correct opportunistic/convenience sampling bias | Predictive spatial maps; mosquito abundance modelling; bite activity modelling | Short temporal window; sampling bias; unvalidated bite reports; misclassification risk; limited climate data; human behavior not included | Participation/sampling bias; Representativeness/generalizability issue; Validation limitation | Accept the Mosquito Alert User Agreement; participation is anonymous. | Mosquito Alert requires acceptance of its user agreement/privacy policy, supports anonymous participation, asks users not to submit personal identifiers, links reports to random user IDs, rounds coordinates by default for privacy, and lets users retain authorship while granting the project reuse rights; shared outputs are generally released under CC-BY/CC0-type licensing. | https://github.com/Mosquito-Alert/sampling_effort_data |
| [2](https://doi.org/10.1109/ACCESS.2021.3079700) | A Deep Convolutional Neural Network for Classification of Aedes Albopictus Mosquitoes | Gereziher Adhane | 2021 | Spain | N/A | Urban | Comparative deep learning study | submitted by residents; "submitted by volunteers"; 6378 images (3364 confirmed Aedes albopictus + 3014 non-tiger) | N/A | N/A | N/A | Data collection; engagement in research or meetings | volunteers | monitor and control disease-carrying mosquitoes | Aedes albopictus (dengue, chikungunya, Zika or yellow fever) | images of tiger mosquitoes and mosquito breeding sites submitted by residents; "images taken with smartphones and digital cameras" Mosquito Alert project | images; "photos submitted"; mosquito images dataset | Hybrid validation (automated + human expert); app-submitted mosquito images; citizen images were initially validated/classified by three expert entomologists, with an additional expert in case of dispute, and this study developed a CNN-based tool to assist/scale image classification; testing accuracy about 94% | GLMM | Not applicable (no quantitative ecological/spatial output); image-classification study focused on automated species identification from citizen-submitted images rather than abundance, hotspot, trend, or other reporting-effort-sensitive outputs | Not applicable (classification performance) | poor image quality; "occluded"; "damaged"; "small sample size" | Data quality issue; Representativeness/generalizability issue | Not stated | Mosquito Alert requires acceptance of its user agreement/privacy policy, supports anonymous participation, asks users not to submit personal identifiers, links reports to random user IDs, rounds coordinates by default for privacy, and lets users retain authorship while granting the project reuse rights; shared outputs are generally released under CC-BY/CC0-type licensing. | <http://www.mosquitoalert.com/en/mosquito-images-dataset> |
| [3](file:///C:\Users\Asus\Desktop\Citizen%20Science\DOI%2010.1186\s12889-017-4289-5) | Camino Verde (The Green Way): evidence-based community mobilisation for dengue control in Nicaragua and Mexico: feasibility study and study protocol for a randomised controlled trial | Neil Andersson | 2017 | Nicaragua; Mexico | N/A | Urban/rural | pragmatic parallel group cluster randomised controlled trial; "feasibility study" | residents in a random sample of 150 clusters (100–120 households each); "children aged 3–9 years" | children aged 3–9 years | N/A | N/A | engagement in research or meetings; monitoring interventions; data collection; community action | community orgs; volunteers | vector control | Dengue | saliva samples; "ELISA"; "entomology evaluators inspect containers"; "flash lights" | text; larvae count; breeding sites | Not applicable; this study did not use citizen-submitted mosquito images/specimens requiring platform-based classification validation; entomological outcomes were assessed by trained evaluators who inspected containers and collected larvae/pupae for laboratory identification | t-test; "non-linear mixed model"; "generalised linear mixed model (GLMM)"; "multiple imputation (Amelia II)"; "intention-to-treat" | Not applicable (no citizen-report-based quantitative output); outcomes were based on a structured cluster RCT with household surveys, saliva samples, and blinded entomological assessments rather than opportunistic citizen reporting, so reporting-effort bias of citizen-submitted observations was not the relevant analytical issue | Risk-factor identification | contamination; "self-selection"; "in-migration and out-migration"; "no randomisation in feasibility study" | Representativeness/generalizability issue | informed consent; "separate informed consent for saliva samples"; "confidentiality"; "Ethical review by University of California, Berkeley; CIET IRB; CIETcanada; Universidad Autónoma de Guerrero" | Not applicable as a platform-level digital policy field. This was a community-based trial rather than an app/platform-based reporting system. | Not available |
| [4](doi:%2010.1136/bmj.h3267) | Evidence based community mobilization for dengue prevention in Nicaragua and Mexico (Camino Verde, the Green Way): cluster randomized controlled trial | Neil Andersson | 2015 | Nicaragua; Mexico | N/A | Urban/rural (community-based) | cluster randomized controlled trial; community intervention | 85,182 residents; 18,838 households; 150 clusters | children aged 3–9 years | N/A | N/A | engagement in research or meetings; monitoring interventions; data collection; community action | Community orgs; volunteers | vector control; education | Dengue | household visits; community meetings; entomology tools; saliva sample collection | text; larvae count; breeding sites; mosquito species; saliva serology | Not applicable; this cluster randomized community-mobilization trial did not rely on citizen-submitted mosquito images/specimens requiring platform-based classification validation; primary vector outcomes came from blinded entomological assessments, with fieldworkers collecting larvae/pupae from containers and laboratories identifying samples | cluster t-test; GLMM; ICC; intention-to-treat; relative risk analysis | Not applicable (no citizen-report-based quantitative output); outcomes were serology, self-reported dengue illness, and entomological indices from structured cluster surveys/assessments rather than opportunistic citizen reporting, so participation-reporting effort bias was not the relevant analytical issue | risk-factor identification | participation bias; generalizability; contamination; limited access; exclusion of wealthy areas | Data quality issue; Recruitment/engagement challenge; Representativeness/generalizability issue | informed consent obtained; confidentiality; IRB approvals | Not applicable as a platform-level digital policy field. This was a community-based intervention trial rather than an app/platform-based reporting system. | Not available |
| [5](https://doi.org/10.1186/s12889-019-7606-3) | Community based integrated vector management for malaria control: lessons from three years' experience (2016-2018) in Botor-Tolay district, southwestern Ethiopia | Abebe Asale | 2019 | Ethiopia | N/A | Urban/rural | Evaluation, longitudinal monitoring | 200 households (community members) | Adults (≥18 household heads) | Male & female household heads | N/A | Monitoring interventions; community engagement; education | Community orgs; local structures | Disease control (malaria prevention) | Malaria | Community mobilization; IRS; LLINs; larviciding; environmental management | Larval counts; mosquito species; breeding sites; survey data | Not applicable; this community-based malaria IVM study did not rely on citizen-submitted mosquito images/specimens requiring platform-based classification validation; entomological monitoring used standard larval dipping and CDC light traps, with mosquitoes morphologically identified by trained staff using taxonomic keys | Descriptive statistics; chi-square; trend analysis | Not applicable (no citizen-report-based quantitative output); outcomes were longitudinal malaria case trends, adult mosquito density, larval abundance, and household survey indicators from structured monitoring rather than opportunistic citizen reporting, so participation/reporting-effort bias was not the relevant analytical issue | Trend analysis (mosquito density, larvae, malaria incidence) | No control group; low net compliance; behavior change slow; confounding | Recruitment/engagement challenge | Written informed consent; ethics approval (KEMRI SERU & district authority) | Not applicable as a platform-level digital policy field. This was a community-based malaria control program rather than an app/platform-based reporting system. | Dataset available upon request |
| [6](http://dx.doi.org/10.3390/su11247012) | Co-Designing a Citizen Science Program for Malaria Control in Rwanda | Domina Asingizwe | 2019 | Rwanda | N/A | Rural | Feasibility; descriptive | 185 participants (workshop attendees), 116 volunteers eN/Aolled | Adults + youth (18–25 group included) | 57% women (overall workshop); mixed | N/A | Data collection; engagement in research or meetings; monitoring interventions | community orgs; volunteers | Surveillance (species identification, nuisance reporting, mosquito collection) | Malaria | smart phone (feedback only; not for data collection), paper forms; handmade CO2-baited trap (plastic bottle + yeast/sugar + torch); buckets (alternative) | text; mosquito species | Not applicable; this paper focused on co-designing the citizen science program rather than validating submitted mosquito observations; participants selected paper forms for reporting mosquito nuisance/confirmed malaria cases and a handmade carbon dioxide-baited trap for mosquito collection, but no implemented species-identification or observation-validation workflow was evaluated in this study | qualitative workshop analysis; no statistical analysis | Not applicable (co-design study; no citizen-report-based surveillance output analyzed); the paper analyzed workshop preferences and design choices for reporting and collection, not mosquito abundance, hotspot, trend, or other participation-sensitive surveillance outputs | No predictive or epidemiological outputs (design-stage only) | participation bias; feasibility constraints; limited phone/SMS access; trap material availability; variation in attendance | Technology/access barrier; Recruitment/engagement challenge; Validation limitation | IRB approved (Approval Notice: 414/CMHS/IRB/2017) | Not applicable as a platform-level digital policy field. This was a locally co-designed community-based citizen science program, not an established app/platform with a standing privacy/user-agreement system. | Not available |
| [7](http://dx.doi.org/10.3390/tropicalmed2030039) | Polisye Kont Moustik: A Culturally Competent Approach to Larval Source Reduction in the Context of Lymphatic Filariasis and Malaria Elimination in Haiti | Kevin Louis Bardosh | 2017 | Haiti | N/A | Urban/Rural | pilot; intervention (community-directed LSM) | ~2500 households; ~10,000 people | N/A | N/A | N/A | Data collection; engagement in research or meetings; monitoring interventions | community orgs; local leaders; volunteers | vector control; Surveillance | Malaria; lymphatic filariasis | manual field maps; no digital tools | larvae count; breeding sites; mosquito species | Not applicable; this paper described a community-directed larval source management pilot and ethnographic/action-research process rather than a citizen-submitted mosquito classification system; surveillance relied on trained “Mosquito Police” conducting larval habitat mapping and surveillance, with supervisory quality checks and later brigadier training for larvicide application, but no formal observation-validation or species-classification workflow for citizen-submitted records was analyzed | qualitative analysis; descriptive assessment | Not applicable (no citizen-report-based surveillance output analyzed); this paper was an ethnographic/action-research reflection on community-directed larval source management, leadership, engagement, cleanup, and larviciding rather than a quantitative analysis of citizen-generated surveillance data such as abundance, hotspots, or temporal trends that would require adjustment for participation/reporting effort | no predictive outputs | participation bias; governance difficulties; social/political constraints; scalability issues | Recruitment/engagement challenge; Validation limitation; Representativeness/generalizability issue | IRB approvals; informed consent | Not applicable as a platform-level digital policy field. This was a locally implemented community-based mosquito control approach rather than an app/platform-based reporting system. | Not available |
| [8](http://doi/10.1111/jvec.12288) | Mosquito traps for urban surveillance: collection efficacy and potential for use by citizen scientists | Mathieu Bazin and Craig R. Williams | 2018 | Australia | N/A | Urban | Descriptive; trap comparison study | N/A | N/A | N/A | N/A | Data collection | Volunteers; convenience recruitment | Surveillance (trap monitoring) | Aedes notoscriptus; Culex quinquefasciatus | Physical traps (BG-Sentinel; BG-GAT); no mobile app | Mosquito species | Expert validation; trap-collected mosquito specimens; volunteers set and retrieved traps, and specimens were identified in the laboratory using taxonomic keys; identification accuracy not reported | Chi-square test; One-way ANOVA; post-hoc Scheffé tests | Not applicable (no citizen-report-based surveillance output analyzed); collection-efficacy and species-composition outputs were based on standardized weekly trap deployments in a controlled trap-comparison study rather than opportunistic citizen reporting, so participation/reporting-effort bias was not the relevant analytical issue | Comparison of trap efficacy; species composition; vector surveillance findings | Participation bias; Generalizability; Data bias | Technology/access barrier; Data quality issue; Validation limitation | Not stated | Not applicable as a platform-level digital policy field. This study evaluated mosquito traps and their usability for citizen scientists, rather than using an established reporting platform with standing privacy/user-agreement terms. | Not available |
| [9](https://doi.org/10.1179/oeh.2007.13.2.188) | An Integrated Ecosystem Approach for Sustainable Prevention and Control of Dengue in Central Havana | Mariano Bonet | 2007 | Cuba | N/A | Urban | Descriptive; Evaluation; Intervention; Case-control; Participatory action research | Community members; 1,179 survey participants; 277 cases & 554 controls | N/A | N/A | N/A | Engagement in research or meetings; Monitoring interventions | Community organizations; neighborhood groups; health councils | Vector control; education | Dengue | Hotspot maps; Risk-factor identification | Breeding sites; Text | Not applicable; this study evaluated an integrated dengue surveillance and community-participation approach rather than a citizen-submitted observation/classification system; mosquito breeding-site detection was conducted through routine surveillance by operarios with quality-control inspection, not through citizen-submitted images/specimens requiring validation | Descriptive statistics; case-control analysis; GIS visualization | Not applicable (no citizen-report-based surveillance output analyzed); outputs were based on integrated environmental, entomological, and clinical-epidemiologic surveillance, GIS mapping, cross-sectional surveys, and a case-control study of households with and without breeding sites rather than opportunistic citizen reporting, so participation/reporting-effort bias was not the relevant analytical issue | Hotspot maps; Risk-factor identification | Generalizability; Participation bias | Recruitment/engagement challenge; Data quality issue | Not stated | Not applicable as a platform-level digital policy field. This was a community-based participatory dengue control approach rather than an app/platform-based reporting system. | Not available |
| [10](https://doi.org/10.1016/j.scitotenv.2019.135349) | Citizen science and smartphone e-entomology enables low-cost upscaling of mosquito surveillance | Larissa Braz Sousa | 2020 | Australia | N/A | Urban (metropolitan setting (Adelaide, South Australia)) | Proof-of-concept; feasibility; evaluation | 126 | Adults (18+) | Mixed | N/A | Data collection, annotating dataset | Open call (crowdfunding + social media + university outreach) | Surveillance (trap monitoring + species identification) | General mosquito monitoring | Web portal; smart phone; email | Image (photo), mosquito species | Expert validation; e-entomology image submissions of trap-collected mosquitoes; citizen scientists photographed collections and emailed images, which were identified and counted remotely by investigators/entomologists; preliminary image-based identification accuracy exceeded 90% for common Australian mosquitoes | ANOVA; Tukey; Simpson index; Shannon-Wiener index; PCA; ANOSIM; nMDS; correlation analysis | Explicit analytical adjustment for effort/bias; abundance and seasonality outputs; mosquito totals were standardized as mosquitoes per trap per day based on elapsed days between citizen submissions, and program comparisons were standardized per trap, although collections remained biased toward container-inhabiting species because of trap type | Trend analysis (seasonality) | Participation bias; data bias (container-breeders overrepresented); generalizability | Spatial bias; Sampling bias; Validation limitation; Technology/access barrier; Recruitment/engagement challenge | Consent obtained from citizen scientists | No single dedicated mosquito-reporting platform with a unified project-level ethics framework was used. Instead, the program relied on third-party services with their own terms/privacy policies, including Chuffed for crowdfunding, Facebook/Meta for group communication, SurveyMonkey for the participant survey, and Tableau/Google-based tools for data display/storage. | <https://public.tableau.com/profile/mozzie.monitors#!/vizhome/MosquitoesinSouthAustralia-GoogleSheets_0/IDSearch> |
| [11](https://doi.org/10.1093/jme/tjae117) | Learning outcomes for participants in citizen science mosquito surveillance | Larissa Braz Sousa | 2024 | Australia | N/A | Urban (residential communities in South Australia and Western Australia) | mixed-methods; evaluation; descriptive | 61 participants (47 Female; 14 male) | 18–80 (mostly 61–70) | 77% female, 23% male | N/A | Data collection; engagement in research or meetings | Open call (via Facebook; university newsletter; community partner | Surveillance (species identification, presence/absence, trap monitoring); education | General mosquito monitoring | smartphone camera; email; iNaturalist; BG-GAT trap | Image (photo); mosquito species | Expert validation; e-entomology image submissions of trap-collected mosquitoes; citizen scientists photographed their catches on the tip card and emailed the images, which were identified by the research team; no formal identification-accuracy metric reported in this paper, although participants improved photo quality and the proportion of identifiable images over time | descriptive statistics; McNemar test; thematic qualitative analysis | Explicit analytical adjustment for effort/bias; abundance and diversity outputs; mosquito abundance was summarized as average mosquitoes collected per trap per elapsed day, active participants and submission frequency were tracked over time, and trap-related species bias plus declining participation were discussed as study limitations | trend analysis (examined seasonal trends, abundance over time) | participation bias; seasonality bias; generalizability; COVID-19 disruptions | Recruitment/engagement challenge; Representativeness/generalizability issue; Sampling bias | Consent obtained from citizen scientists; privacy | No single dedicated mosquito-reporting platform with a unified project-level ethics framework was used. Mozzie Monitors was a university-run program using study-specific consent and human ethics approval procedures, with communication and data collection handled through general university/third-party tools rather than a standalone app/web platform with standard public user-agreement/privacy terms. | Not available |
| [12](https://doi.org/10.3390/ijerph19106337) | Citizen Science Mosquito Surveillance by Ad Hoc Observation Using the iNaturalist Platform | Larissa Braz Sousa | 2022 | Australia | N/A | Urban (nationwide online community) | Descriptive (The study analyzes iNaturalist submissions and user behavior) | >500 observers (ad hoc community participants) | 18–80 | 27% female, 69% male, Prefer not to say (2%), Other (2%) | N/A | Data collection; annotating dataset | Open call (iNaturalist platform; public upload) | Surveillance (species identification) | General mosquito monitoring | Mobile apps; Web portal (iNaturalist) | Image (photo); mosquito species | Community / participant-assisted validation; ad hoc iNaturalist photo observations of mosquitoes; identifications were generated through the iNaturalist community/crowdsourcing system, with expert medical entomologists also validating Mozzie Monitors mosquito identifications; research-grade status required date, geolocation, media, and agreement by at least two-thirds of identifiers | descriptive statistics; time-series analysis; community composition analysis (NMDS with Bray–Curtis); dissimilarity analysis | Acknowledged as limitation only; species distribution, seasonality, and community-composition outputs; the study discussed strong species, location, observer, and participation biases qualitatively, including over-representation of large-bodied/colorful mosquitoes and concentration of observations around major cities, but did not apply an explicit analytical correction for reporting effort | Trend analysis (Seasonality curves, Geographic hotspots, Species distribution patterns) | participation bias; data bias (Species bias, location bias, observation bias, over-representation of certain species) | Spatial bias; Sampling bias; Data quality issue; Recruitment/engagement challenge | Consent obtained from citizen scientists; privacy (GPS obfuscation option); data ownership (iNaturalist terms) | iNaturalist is governed by its Terms of Use and Privacy Policy; users must accept these terms when using the platform, users retain ownership of submitted content while licensing it on the platform, and observation data can include time/date/location and be publicly shared unless the user chooses to hide/obscure sensitive information. | https://www.inaturalist.org/projects/mozzie-monitors-australia |
| [13](https://doi.org/10.1371/journal.pntd.0009698) | Aedes albopictus bionomics data collection by citizen participation on Procida Island, a promising Mediterranean site for the assessment of innovative and community-based integrated pest management methods | Beniamino Caputo | 2021 | Italy | N/A | Urban (entire island), community-wide | descriptive; intervention (community-based monitoring & MRR) | ~300 residents involved; 12 ovitrap volunteers; 99 households; 20 families in MRR | N/A | N/A | N/A | Data collection; engagement in research or meetings | Community orgs; volunteers (municipality selected volunteers, community families agreed to participate) | Surveillance (trap monitoring, presence/absence), education, vector control (monitoring mosquito population, participating in educational events, supporting future vector-control planning) | General mosquito monitoring (Aedes albopictus) | None (ovitraps, BG-Sentinel traps, aspirators) | larvae count; mosquito species (egg counts, adult traps) | Expert validation; citizen-managed ovitrap egg collections and adult recaptures; volunteers were trained by expert operators to manage ovitraps and deliver germination paper strips, eggs were counted under a stereomicroscope, and field-collected mosquitoes were identified using morphological keys; no formal identification-accuracy metric reported | Generalized Linear additive Mixed Models (GAMM); INLA; Kriging; GLM; Fisher-Ford population estimates | Not applicable (standardized citizen-assisted monitoring rather than opportunistic reporting); temporal, spatial, and population-size outputs were based on fixed weekly ovitrap monitoring, structured spatial sampling, and mark-release-recapture analyses using GAMM, kriging, and population-size models, so participation/reporting-effort bias was not the relevant analytical issue | Spatial predictions; Trend analysis | participation bias; generalizability (Variation in ovitrap data, Limited to one island, Citizens differ in involvement levels) | Recruitment/engagement challenge; Data quality issue | Consent obtained from citizen scientists; privacy; data ownership | Not applicable as a platform-level digital policy field. This was a locally organized community-based monitoring project using study-specific ethics approval, municipal approval, and informed consent, rather than a standing app/platform privacy and user-agreement framework. | All data available in manuscript & Supporting Information |
| [14](https://doi.org/10.3390/ijerph17217872) | ZanzaMapp: A Scalable Citizen Science Tool to Monitor Perception of Mosquito Abundance and Nuisance in Italy and Beyond | Beniamino Caputo and Mattia Manica | 2020 | Italy | N/A | Urban (nationwide Italian communities) | descriptive; evaluation | 13669 users; 36867 records | N/A | N/A | N/A | Data collection (citizens submitted observations via the app) | open call (Users joined because of TV, news, radio, university, municipal outreach) | Surveillance (presence/absence); Education | General mosquito monitoring (Aedes albopictus) | mobile app (Android & iOS); web portal; smartphone, GPS, Zanzamapp | text | Not validated / not described; citizen records were self-reported perceptions of mosquito abundance/nuisance submitted through a simple questionnaire without photo or specimen authentication; the study included a first small-scale field validation of app outputs against Human Landing Collections (HLC) of *Aedes albopictus* in Procida | descriptive statistics; linear regression; GAM; spatial analysis | External validation / benchmarking only; perceived mosquito abundance and nuisance outputs; app records were benchmarked against HLC data in Procida, while major participation/reporting biases (advertising effects, unequal user engagement, population-density dependence, and bias toward positive winter records) were analyzed and discussed but not directly corrected through an explicit effort-adjustment model | hotspot maps; trend analysis; spatial predictions | data bias; perception bias; participation bias; misidentification; generalizability | Recruitment/engagement challenge; Sampling bias; Data quality issue; Validation limitation | no explicit consent; anonymized data; privacy maintained | ZanzaMapp is governed by its legal/privacy notice; use of the app implies acceptance of the terms, it collects geolocation plus device ID/IP for functionality, and states that these data may be stored and used in anonymous form for scientific and statistical analyses. It also states that third-party contributed content remains the author’s property, including the right to request deletion. | zanzamapp.it |
| [15](https://doi.org/10.5334/cstp.616) | Citizen Science as an Approach for Responding to the Threat of Anopheles stephensi in Africa | Ryan M. Carney | 2023 | Africa (multi-country) | N/A | Urban | Descriptive (conceptual/review) | N/A | N/A | N/A | N/A | Data collection; annotating dataset; engagement in community campaigns; monitoring interventions | open call; community organizations; volunteers | Surveillance (species identification, presence/absence); education | Malaria | Mobile apps (Mosquito Alert; GLOBE Observer; iNaturalist); Web portal (GMOD; mosquitoID.org); GPS; smart phone | Image (photo); text; larva count; breeding sites; mosquito species | Hybrid validation (automated + human expert); smartphone photos of larval/adult mosquitoes submitted through citizen science platforms; Mosquito Alert uses expert communities/volunteer entomologists to validate and annotate photos and is increasingly using AI to classify images, with human experts still revising results as needed; additional AI tools were described for identifying *An. stephensi* and other mosquito traits, with further testing through molecular validation | AI-based image classification (no statistical analysis) | Not applicable (conceptual/essay paper; no citizen-report-based quantitative output analyzed); the paper proposes citizen science, AI tools, app deployment, and surveillance mobilization for *Anopheles stephensi* detection, but it does not present a quantitative ecological analysis of abundance, hotspots, temporal trends, or risk outputs requiring explicit correction for participation/reporting effort | Risk models; Trend analysis; Spatial predictions; Risk-factor identification | data bias (dependent on citizen smartphone access & image quality); algorithm limitations (AI still in beta, may produce false positives); participation bias (uneven geographic reporting; urban-biased); generalizability (tools validated mostly outside Africa; early-stage for An. stephensi) | Technology/access barrier; Validation limitation; Recruitment/engagement challenge | privacy; data ownership | No single shared platform policy applies. Instead, each tool has its own terms/privacy framework: GLOBE Observer uses user accounts with public screen names rather than public personal identifiers and promotes open data sharing; Mosquito Alert requires acceptance of its user agreement/privacy policy and supports anonymous participation; iNaturalist operates under its Terms of Use/Privacy Policy and lets users retain ownership while choosing licenses for their content. | [https://mosquitodashboard.org](https://mosquitodashboard.org/) |
| [16](https://doi.org/10.3390/insects13080675) | Integrating Global Citizen Science Platforms to Enable Next-Generation Surveillance of Invasive and Vector Mosquitoes | Ryan M. Carney | 2022 | USA | Florida; global | Urban/rural (global multi-country) | Descriptive (a methodological & descriptive integration study) | N/A | N/A | N/A | N/A | Data collection; annotating dataset; monitoring interventions (They submit photos, larvae counts, habitats; experts annotate; AI monitors patterns). | open call (Global media campaigns + app availability = open recruitment.) | Surveillance (species identification, presence/absence, trap monitoring), education, vector control | General mosquito monitoring | Mobile apps (Mosquito Alert, iNaturalist, GLOBE Observer); Web dashboards; smartphone; GPS; sensors (clip-on macro lens) | Image (photo), text, larve count, breeding sites, mosquito species | Hybrid validation (automated + human expert); cross-platform citizen-science mosquito images and habitat records; Mosquito Alert adult photos were validated by entomologists and was moving toward AI with a human-in-the-loop approach, GLOBE Mosquito Habitat Mapper larval records required expert validation and manual photo screening, and iNaturalist observations relied on community identification to reach Research Grade; pooled images were also used to train AI species/anatomy models | Descriptive statistics; data harmonization; AI model training & validation (CNN, Mask-RCNN); confusion matrices; accuracy metrics | External validation / benchmarking only; presence/early-detection and habitat-model-validation outputs; citizen-science observations were used to validate a trap-based *Aedes aegypti* habitat model and complement trap/risk-mapping systems, while observer bias was explicitly noted through higher population density around most citizen-science locations, but no formal analytical correction for participation/reporting effort was applied | Hotspot maps, Trend analysis, Spatial predictions, Risk-factor identification | Data bias, algorithm limitations, participation bias, generalizability (population-density bias, AI constraints, needing validation, uneven global coverage) | Sampling bias; Spatial bias; Recruitment/engagement challenge; Validation limitation; Technology/access barrier | Consent obtained from citizen scientists, privacy, data ownership | No single shared platform policy applies. Instead, each tool has its own terms/privacy framework: Mosquito Alert requires acceptance of its user agreement/privacy policy and supports anonymous participation; GLOBE Observer uses account-based participation under NASA/GLOBE privacy rules, collects limited account information, and screens photos for faces/text; iNaturalist operates under its Terms of Use/Privacy Policy and lets users retain ownership while licensing submitted content. | https://mosquitodashboard.org/ |
| [17](https://doi.org/10.3389/fenvs.2021.682669) | The STEM Enhancement in Earth Science “Mosquito Mappers” Virtual Internship: Outcomes of Place-Based Engagement with Citizen Science | Holly Cho | 2021 | USA | nationwide | Urban/rural | Descriptive, evaluation (an educational program evaluation) | 113 interns eN/Aolled; 60 completed | 15–18 years | 30 females, 27 males, out of 60 | Reported in Table 1 (various categories, not tied individually) | Data collection; annotating dataset; engagement in research or meetings; data analysis (collected data using mobile app, annotated data, participated in mentor meetings, analyzed data) | community orgs (Students applied and were selected via a national NASA-linked program) | Education (learning + local mosquito habitat documentation) | General mosquito monitoring | Mobile apps (GLOBE Observer); GPS; smart phone | Image (photo) (Geo-tagged images; habitat photos; land cover photos; mosquito habitat attributes) | Not applicable; this paper evaluated educational and career-orientation outcomes of a virtual high-school internship using the GLOBE Observer app, rather than the classification or validation of citizen-submitted mosquito observations; interns used Mosquito Habitat Mapper and Land Cover as data-collection tools within local 3 km² areas of interest for research projects | Wilcoxon signed-rank tests; descriptive statistics; thematic qualitative analysis | Not applicable (education/internship outcomes study; no citizen-report-based ecological or surveillance output analyzed); analyses examined knowledge gains, career interest, motivation, and program experiences using quizzes, Likert-scale surveys, and thematic analysis, rather than abundance, hotspot, trend, or risk outputs requiring adjustment for participation/reporting effort | None (study focuses on educational outcomes, not epidemiological modeling) | Participation bias; generalizability | Recruitment/engagement challenge | Consent obtained from citizen scientists | GLOBE Observer uses account-based participation under NASA/GLOBE privacy rules; users register with an email address and country, public data are linked to a screen name rather than personal identifiers, email addresses are not publicly displayed, children under 13 should participate under adult/school supervision, and photos with faces or personal identifiers are screened out. | globe.gov/globe-data |
| [18](https://doi.org/10.5334/cstp.679) | Citizen Science for Enhanced Dengue Vector Surveillance in Solomon Islands: A Methods Paper | Adam Craig | 2024 | Solomon Islands | N/A | urban/rural | pilot, descriptive, feasibility, methods | 340 students + 16 scholars + 18 schools | ~16 years | N/A | N/A | Data collection, monitoring interventions, engagement in research or meetings | community orgs, volunteers | Surveillance (trap monitoring, mosquito species identification), education, vector control | Dengue | Mobile apps (Meditrack), Web portal (Tupaia), GPS, smart phone, Sensor (ovitraps) | larve count, mosquito species, breeding sites, text | Expert validation; citizen scientists (school students) built and deployed ovitraps and returned egg strips, then mosquitoes were reared to adults and identified to species using morphological methods and an adult identification key by SINU scholars under the supervision of a medical entomologist; the earlier pilot phase also reported 94% agreement between participant and entomologist assessments | Descriptive statistics; POI (positive ovitrap index); MET (mean eggs per trap); MMT_S (mean mosquitoes per trap by species) | Not applicable (standardized citizen-assisted monitoring rather than opportunistic reporting); presence and abundance outputs were derived from a structured school-based ovitrap network with standardized deployment and collection, and analyzed using entomological indices (POI, MET, MMT_S) plus descriptive spatial-temporal analysis, so participation/reporting-effort bias of ad hoc citizen observations was not the relevant analytical issue | Hotspot maps (distribution maps) | participation bias, generalizability, data bias | Sampling bias; Technology/access barrier; Recruitment/engagement challenge; Representativeness/generalizability issue | Consent obtained from citizen scientists (students informed), privacy; no formal ethics review required | No clear public platform-level ethics framework was reported. Tupaia/Meditrack was used as a custom project data system with user-specific access rights and cloud-based storage, so ethics/governance appear to be mainly project-led rather than platform-led. | All data are included in the manuscript. |
| [19](https://doi.org/10.1186/s12889-021-10493-6) | Citizen science as a tool for arboviral vector surveillance in a resourced-constrained setting: results of a pilot study in Honiara, Solomon Islands, 2019 | Adam T. Craig | 2021 | Solomon Islands | N/A | Urban (Honiara city) | Feasibility; pilot | 13 | 20–58 | 8 Males and 5 Females | N/A | Data collection (Participants only trapped mosquitoes, identified species, and sent counts) | Volunteers (snowball sampling) | Surveillance (species identification, trap monitoring) (Participants only trapped and identified mosquitoes) | Dengue; Zika; Chikungunya | Smart phone(SMS reporting); physical traps (BG-Sentinel II) | Mosquito species; larval counts; text | Expert validation; trap-collected adult mosquitoes; participants identified catches using a magnifying glass and pictorial identification card, and specimens were then independently reviewed blind by an entomologist; agreement between participant and entomologist identification was 94% | Descriptive statistics; Mann–Whitney U test; agreement validation between participant and entomologist; median and interquartile range calculations | Not applicable (standardized citizen-assisted trap surveillance rather than opportunistic reporting); spatial-temporal and count outputs were based on weekly household trap catches and SMS reporting over an 8-week period, and the study assessed participation stability/completeness as a feasibility outcome rather than applying an analytical correction for reporting-effort bias | Spatial distribution (mapped mosquito presence); Trend analysis | participation bias (barriers that reduce ongoing engagement , unstable electricity, cost issues, forgetting, being too busy, poor lighting, trap inconvenience, selection bias (snowball), motivation issues, technology barriers); Data bias (inaccuracies or incomplete reports due to electricity outages, inability to send SMS, environment not suitable for identification, trap disturbances); Generalizability (small sample, limited region (Honiara only), cannot generalize to all Solomon Islands or Pacific populations) | Recruitment/engagement challenge; Technology/access barrier; Validation limitation; Representativeness/generalizability issue | Consent obtained from citizen scientists | Not applicable as a platform-level ethics field. This was a study-specific surveillance setup, not an established app/platform; ethics were handled at the project level through written consent, ethics approvals, and secure data storage. | Not publicly available (data restricted by ethics; available upon request) |
| [20](https://doi.org/10.3390/insects16020128) | Online Crowdsourced Data from iNaturalist Can Assist Monitoring of Invasive Mosquitoes | Benjamin Cull | 2025 | Europe & neighbouring countries (62 countries) | N/A | Urban | descriptive | N/A | N/A | N/A | N/A | Data collection ((Public submits mosquito photos) | volunteers (iNaturalist is open-access and relies on voluntary public participation) | Surveillance (species identification; presence/absence) ((Study uses iNaturalist photos to detect mosquitoes, new areas of spread, and seasonal patterns)) | General mosquito monitoring | Web portal; Mobile app; Smartphone (iNaturalist): operates via mobile app + web platform, using smartphone photos) | Image (photo); mosquito species | Expert validation; iNaturalist mosquito photo observations; all Culicidae records were downloaded and images were re-identified by the author using mosquito identification keys, with invasive Aedes identifications categorized as confirmed or suspected; community-generated iNaturalist identifications were then compared against expert identifications, with accuracy of 87.6% for *Aedes albopictus*, 81.3% for *Aedes aegypti*, and 60.3% for combined *Aedes japonicus*/*koreicus* | Descriptive statistics; mapping; expert validation; seasonal trend analysis | Acknowledged as limitation only; invasive-species distribution and seasonal-occurrence outputs; observations were mapped and compared with VectorNet records and monthly activity patterns, but collection bias toward high-population-density areas and variable image quality/identification quality were discussed qualitatively rather than corrected with an explicit effort-adjustment model | Spatial distribution maps; Trend analysis; Species distribution comparison (Outputs include maps comparing iNaturalist vs VectorNet and seasonal activity plots) | Data bias; generalizability; participation bias (Bias due to urban concentration, uneven participation, misidentification, incomplete coverage) | Spatial bias; Data quality issue; Validation limitation | Not stated | iNaturalist is governed by its Terms of Use and Privacy Policy; users accept these when using the platform, retain ownership of their submitted content, license observations on the platform, and can choose how location/time-linked observations are shared or removed. | [https://www.mdpi.com/article/10.3390/insects16020128/s1: Supplementary Table S1 (raw iNaturalist Culicidae dataset)](https://www.mdpi.com/article/10.3390/insects16020128/s1:%20Supplementary%20Table%20S1%20(raw%20iNaturalist%20Culicidae%20dataset)) |
| [21](https://doi.org/10.1016/s0001-706x(01)00178-4) | Control of malaria vectors with the insect growth regulator pyriproxyfen in a gem-mining area in Sri Lanka | A.M.G.M. Yapabandara | 2001 | Sri Lanka | N/A | Urban/rural | intervention (treated vs control villages) | | N/A | N/A | N/A | Data collection (volunteers located pits and breeding sites) | community orgs (volunteers recruited through village meetings) | Surveillance (presence/absence, trap monitoring) | Malaria | Sensor (physical larval monitoring tools used) | mosquito species and larval counts (adult and larval surveillance) | Not applicable; this malaria vector-control trial did not rely on citizen-submitted mosquito images, specimens, or records requiring a platform-based validation workflow; local volunteers mainly supported community participation activities such as helping locate breeding sites and supporting intervention implementation, while adult mosquito populations were monitored using standard entomological collection methods | paired t-tests; Mantel-Haenszel chi-square; percent change estimates | Not applicable (structured intervention and entomological monitoring study rather than opportunistic citizen reporting); adult mosquito abundance, malaria incidence, and parasitaemia outcomes were derived from standardized vector sampling, clinic-based passive case detection, and mass blood surveys, so participation/reporting-effort bias from ad hoc citizen observations was not the relevant analytical issue | Risk-factor identification (epidemiologic reduction patterns) | generalizability; data bias; participation bias (population movement and volunteer dependence) | Representativeness/generalizability issue | Not stated | Not applicable as a platform-level ethics field. This was a locally organized community-based intervention, not an established digital platform with standing user-agreement, privacy, or data-ownership policies. | Not publicly available |
| [22](https://doi.org/10.52707/1081-1710-49.2.r12) | iNaturalist community observations provide valuable data on human-mosquito encounters | Benjamin Cull | 2024 | UK & Ireland | N/A | Urban/rural | descriptive (observational study) | N/A | N/A | N/A | N/A | Data collection (Citizens only upload photos; no analysis, no meetings.) | volunteers; open call (Anyone on iNaturalist could upload; no targeted recruitment) | Surveillance (species identification, presence/absence, seasonal monitoring) (All citizen contributions are photos used for species identification and mapping) | General mosquito monitoring | Mobile apps; Web portal (iNaturalist) | Image (photo), text, mosquito species | Expert validation; iNaturalist mosquito photo observations; each observation was individually assessed by a medical entomologist using keys to British and European mosquitoes, and expert identification overrode conflicting iNaturalist community identifications; the paper also describes iNaturalist research-grade/community agreement, but did not rely on that alone; adult observations were identifiable to species/species-complex in 73.4% of cases, whereas immature-stage identification was much lower (3.4%) | Descriptive statistics; chi-square test; cross-correlation | Bias examined / benchmarked but not directly corrected; human-mosquito encounter, species composition, seasonality, distribution, urban-association, and biting-behavior outputs; iNaturalist records were compared with long-term NBN and Mosquito Recording Scheme datasets and linked to land-cover data, while spatial reporting bias toward high-population-density urban areas and variation in photo/user expertise were explicitly discussed, but no formal analytical correction for participation/reporting effort was applied | Trend analysis; Spatial predictions (distribution mapping & seasonal patterns) | data bias; participation bias; generalizability (urban bias, uneven reporting for data bias, more uploads in high-population areas for participation bias, rural/rare species underrepresented for generalizability) | Spatial bias; Data quality issue; Validation limitation | Consent obtained via iNaturalist platform; privacy via location-obscuring; data ownership retained by users | iNaturalist is governed by its Terms of Use and Privacy Policy; users accept these terms when using the platform, retain ownership of submitted content while licensing it on the platform, and observation data can include date/time/location and be publicly shared unless the user chooses to hide location details. | <https://www.inaturalist.org/projects/mozzie-monitors-uk-ireland> |
| [23](https://doi.org/10.1186/s41256-023-00298-y) | What incentives encourage local communities to collect and upload mosquito sound data by using smartphones? A mixed methods study in Tanzania | Rinita Dam | 2023 | Tanzania | N/A | Rural | descriptive; evaluation. Mixed methods (qualitative + quantitative empirical trial + feedback survey) | 148 | 18–69 | Male & Female | N/A | Data collection (participants used HumBug sensors to collect mosquito sound data) | Community orgs (participants recruited by village leaders using purposive + snowball sampling) | Education (learning about mosquito species), Surveillance (presence/absence, via acoustic recording) | Malaria (focus on malaria vector species) | Smart phone, Sensor (HumBug acoustic sensor via MozzWear app; SMS reminders used) | text; mosquito species, Audio data (mosquito flight-tone recordings), text (survey & qualitative responses) | Automated classification; mosquito flight-tone audio recordings collected via smartphones; the HumBug sensor records acoustic signatures with time and location and sends them to a server where algorithms identify mosquito species from their characteristic sounds; no validation-accuracy metric or human verification workflow was reported in this paper | Descriptive statistics, Two-sided z-test (binomial), Thematic qualitative analysis (Statistical analysis: descriptive + evaluation) | Not applicable (participation/incentives study rather than ecological surveillance-output analysis); the main outcome was whether participants switched on the HumBug sensor and uploaded mosquito sound data, with binary upload activity compared across trial groups, rather than abundance, hotspot, temporal-trend, or risk outputs requiring correction for participation/reporting effort | Trend analysis (participation patterns) | Participation bias (control group uploaded more), generalizability (village-level randomization), data bias (charging issues) | Technology/access barrier; Recruitment/engagement challenge | Consent obtained (written informed consent; ethics approvals from Oxford, NIMR, IHI-IRB) (Ethics: consent obtained) | HumBug/MozzWear has a privacy-focused project policy: the app records environmental sound, uses real-time voice-activity detection, automatically deletes clips containing human voice, and deployment areas should be clearly signposted; the HumBug website also follows the University of Oxford privacy policy. | Not publicly available (dataset protected; available upon request) |
| [24](https://doi.org/10.1186/s12889-022-14792-4) | Community efforts to monitor and manage Aedes mosquitoes (Diptera: Culicidae) with ovitraps and litter reduction in east Tennessee | C. A. Day and R. T. Trout Fryxell | 2022 | USA | Tennessee | urban/rural (Urban/suburban school campuses, community neighborhoods) | feasibility, evaluation, descriptive, intervention (Descriptive + community-engaged surveillance + BACI intervention evaluation) | 146 cleanup volunteers (17 schools (2019); 15 schools (2020); 13 schools (2021)) | N/A | N/A | N/A | Data collection, engagement in research or meetings, data analysis, monitoring interventions | open call, community orgs, volunteers (Volunteers; educator recruitment through workshops; community organization involvement) | Surveillance (presence/absence), education, vector control (purpose: species ID, presence/absence monitoring, classroom learning, cleanup) | General mosquito monitoring (La Crosse virus risk indirectly (mosquito-borne disease covered)) | Ovitraps; egg papers; mosquito rearing equipment | mosquito species (Egg counts; species ID; larval/adult emergence data) | Expert validation; citizen-/school-collected ovitrap egg papers; educators and students collected Aedes eggs with standardized ovitraps, and UTK later counted eggs under a dissecting microscope, reared mosquitoes from egg to adulthood, and identified adults to sex and species using microscopic characters; the study also explicitly evaluated whether school-collected data matched UTK-collected data | GLMM; BACI analysis; descriptive statistics; boxplots (statistical analysis) | Not applicable (standardized citizen-assisted monitoring rather than opportunistic reporting); egg-abundance and cleanup-intervention outputs were based on structured ovitrap deployments with fixed weekly collection periods, and analyses used UTK comparison datasets plus a BACI design rather than ad hoc citizen submissions requiring correction for participation/reporting effort | Trend analysis (intervention impact assessment (predictive/epidemiological outputs)) | Data bias; rearing limitations; participation bias; trap placement bias; generalizability | Recruitment/engagement challenge; Data quality issue; Validation limitation | IRB approval and educator consent (UTK-IRB-19-05046-XP) (ethics) | Not applicable as a platform-level ethics field. This was a community/school-based program, not an established app or web platform with standing user-agreement, privacy, or data-ownership policies; ethics were handled at the project level through IRB approval and participant consent. | https://megabitess-tga.hub.arcgis.com/ |
| [25](https://doi.org/10.1186/s13071-021-04874-4) | At the tip of an iceberg: citizen science and active surveillance collaborating to broaden the known distribution of Aedes japonicus in Spain | Roger Eritja | 2021 | Spain | N/A | urban/rural | Descriptive, surveillance (multi-source monitoring design) | N/A | N/A | N/A | N/A | Data collection (citizens submitted reports/photos; some helped collect larvae) | Open call (voluntary submissions via the Mosquito Alert app) | Surveillance (species identification & presence/absence) | General mosquito monitoring (invasive Aedes japonicus) | Mobile apps, Web portal, GPS, smartphone (Mosquito Alert platform) | Image (photo), text, mosquito species (submitted photos + field samples) | Expert validation with field confirmation; Mosquito Alert citizen-submitted geolocated adult-mosquito photos; images were first validated online by experienced Mosquito Alert entomologists using predefined classification criteria and likelihood-based tagging, and reports from previously unknown areas then required field confirmation through active sampling by the Ministry of Health, local authorities, and academic teams; additional reports were sometimes kept as probable/definite photo-based records without field confirmation when resources or participant follow-up were lacking; no formal accuracy metric reported | None (descriptive study; no statistical tests) | Presence/early-detection surveillance; sampling effort characterized but not formally corrected; the platform used UUIDs/background tracking to estimate spatio-temporal variation in sampling effort and noted declining engagement over time, but the study’s main outputs were species detection and range expansion confirmed through follow-up field surveillance rather than abundance, hotspot, or trend models requiring explicit reporting-effort correction | Spatial predictions (mapping of expanded distribution from <900 km² to >7000 km²) | Participation bias; generalizability (uneven reporting; limited field confirmation) | Validation limitation | Not stated | Mosquito Alert requires acceptance of its user agreement and privacy policy, supports anonymous participation, asks users not to include personal information, links reports to a randomly generated user ID, and allows submitted data/images to be shared under project licensing/open-data terms. | https://www.mosquitoalert.com (public dataset map) |
| [26](https://doi.org/10.1038/s41598-017-12652-5) | Direct Evidence of Adult Aedes albopictus Dispersal by Car | Roger Eritja | 2017 | Spain | Not applicable (not in United States) | Urban | Descriptive observational study (systematically sampling cars and analysing observations) (methods describe observational sampling of 770 cars) | 770 cars sampled (drivers participated by volunteer basis; cars are the sampled units) | N/A | N/A | N/A | Data collection; engagement in research or meetings (citizen scientists reported mosquitoes in cars; researchers vacuum-sampled vehicles) (citizen alerts and field sampling both contribute) | Volunteers (drivers voluntarily agreed to car inspection; citizen scientists voluntarily submitted app reports) | Surveillance (presence of mosquitoes in cars; passive transport monitoring) (goal was to quantify car-mediated dispersal) | Aedes albopictus (focus species and competent vector for dengue, Zika, chikungunya) (explicitly stated early in paper) | Web portal; mobile app (Mosquito Alert app + web-based platform) | Image (photo) and text (photos and notes submitted through Mosquito Alert platform) | Expert validation; Mosquito Alert citizen-submitted tiger-mosquito reports/photos used to corroborate car-mediated dispersal; citizen scientists’ photographs were validated by expert entomologists, and the field component also involved species identification of mosquitoes captured from sampled cars; no formal photo-validation accuracy metric reported in this paper | Bayesian logistic regression, posterior predictive modelling, regression with priors, sensitivity analysis (statistical modelling described extensively) (logistic regression models described) | Explicit analytical handling using an effort-adjusted citizen-science proxy; car-transport probability and inter-province transfer outputs; the study combined direct car sampling with Mosquito Alert–based tiger-mosquito alert probabilities treated as a proxy for mosquito prevalence, and those probabilities were estimated conditional on sampling effort rather than from raw citizen-report counts alone | Spatial predictions; trend analysis (predicting mosquito flows across provinces; estimating probability of car-transport) (flow modelling and risk estimation shown) | Generalizability ; data bias (low number of positive detections, rare event bias, uncertainty in model estimates) | Validation limitation; Data quality issue | Consent obtained and privacy (drivers participated anonymously; no personal data recorded; Mosquito Alert anonymizes locations) | Mosquito Alert requires acceptance of its user agreement and privacy policy, supports anonymous participation, asks users not to include personal information, links reports to a randomly generated user ID, and allows submitted images/data to be shared under project licensing and open-data terms. | <http://doi.org/10.5281/zenodo.838803> |
| [27](https://doi.org/10.1186/s13071-019-3317-y) | First detection of Aedes japonicus in Spain: an unexpected finding triggered by citizen science | Roger Eritja | 2019 | Spain | N/A | Rural | Descriptive (species detection and field confirmation) | N/A | N/A | N/A | N/A | Data collection (citizens submitted photos, larvae, adults enabling detection) | Open call (citizens voluntarily submitted reports through Mosquito Alert) | Surveillance, species identification (citizen reports triggered investigation) | General mosquito monitoring; Aedes japonicus; WNV relevance only | Mobile apps; Web portal; smartphone (Mosquito Alert app) | Image (photo); larvae; adult specimens; breeding-site observations | Expert validation with participant follow-up and field confirmation; Mosquito Alert citizen-submitted georeferenced photos of adult mosquitoes; all images were independently evaluated by three experts with coordinator consensus when needed, the original participant was contacted through the app and provided additional larvae and adults, the species was then confirmed morphologically and through a field mission, with one adult also confirmed by cox1 sequencing; no formal validation-accuracy metric reported | None | Presence/early-detection surveillance; no formal effort correction reported; citizen reports were used to trigger confirmation and broaden evidence on distribution, and the study explicitly noted that media outreach sharply increased participation in Asturias, but the main output was species detection/delimitation rather than abundance, hotspot, or temporal-trend estimation requiring analytical correction for reporting effort | None | Generalizability (limited geographic area of detection); participation bias (citizen reports varied by media exposure) (paper notes increased reports after media and limited detection region) | Validation limitation; Representativeness/generalizability issue; Recruitment/engagement challenge | Privacy (citizens remained anonymous unless voluntarily revealing identity) | Mosquito Alert requires agreement to its user agreement and privacy policy, supports anonymous participation, asks users not to include personal information, links reports to a randomly generated user ID, and states that shared works/images may be released publicly under project licensing terms, while parts of resulting databases may be shared under CC0. | [http://www.mosquitoalert.com](http://www.mosquitoalert.com/) |
| [28](https://doi.org/10.3390/insects16090904) | Integrating Citizen Science and Field Sampling into Next-Generation Early-Warning Systems for Vector Surveillance: Twenty Years of Municipal Detections of Aedes Invasive Mosquito Species in Spain | Roger Eritja | 2025 | Spain | N/A | Urban/rural | Descriptive, retrospective surveillance analysis (pilot, feasibility, evaluation, descriptive, intervention ) | N/A | N/A | N/A | N/A | Data collection, engagement in surveillance, annotating dataset, monitoring interventions (citizens submit mosquito photos; experts validate; users respond to national alerts, high multi-level involvement typical of digital citizen science) | Open call, community orgs, volunteers (Mosquito Alert relies on voluntary downloads and participation) | Surveillance (species identification, early detection), education, vector control | General mosquito mointoring (Aedes albopictus, Aedes aegypti, and Aedes japonicus) | Mobile apps, Web portal, Sensor, GPS, smart phone (Mosquito Alert is the primary digital tool) | Image (photo), text, larva count, breeding sites, mosquito species | Hybrid validation (AI + expert); Mosquito Alert geolocated photo reports of adult mosquitoes; each incoming report received an initial AI classification and was then evaluated by three expert culicid entomologists, with supervisor review/consensus, and reports were classified into likelihood categories such as “probable” or “confirmed”; no formal validation-accuracy metric was newly reported in this paper | Descriptive statistics, spatiotemporal mapping, geometric centroids (GIS), permutation tests, t-tests, ANOVA, distance analysis (statistical analysis methods used for comparison of strategies) | Presence/early-detection surveillance; sampling-effort modeling acknowledged but not applied in this study; the paper states that Mosquito Alert includes procedures to estimate user sampling effort and cancel opportunistic sampling in predictive models, but for this study the effort model was not used because the aim was to compile first municipal detections and compare detection patterns, not to estimate abundance, hotspots, or temporal trends from raw citizen reports | Hotspot maps, Trend analysis, Spatial predictions, Risk-factor identification | Participation bias, data bias, mislocation of reports, inconsistent field metadata, variability in citizen engagement | Sampling bias; Spatial bias; Validation limitation; Data quality issue; Recruitment/engagement challenge; Representativeness/generalizability issue | Consent obtained implicitly through app terms; complete anonymity; privacy protected; open data licensing (Mosquito Alert uses CC0 licensing and stores no personal identifiers) | Mosquito Alert requires acceptance of its user agreement and privacy policy, supports anonymous participation, asks users not to include personal information, links observations to a randomly generated user ID, and states that data are made available under open-data terms, including CC0 for part of the database. | <https://doi.org/10.5281/zenodo.15869762> |
| [29](https://doi.org/10.52707/1081-1710-47.1.117) | Utilizing citizen science to model the distribution of Aedes aegypti in West Africa | Freeman EA | 2022 | West Africa | N/A | Urban/rural | Descriptive ecological modeling study (compares data sources and models species distribution) | 3607 observations from GLOBE and 55 literature-derived Ae aegypti points | N/A | N/A | N/A | Data collection (citizen scientists recorded larval habitats, eggs, larvae, adults via app, active data collection) | Open call; volunteers (GLOBE app is openly accessible and used by schools and individuals) | Surveillance for species distribution modeling (collecting habitat and species presence to improve distribution models) | General mosquito mointoring (Dengue, Zika, chikungunya (the three major Aedes-borne arboviruses referenced as public health threats in West Africa)) | Mobile app and web portal (GLOBE Observer mobile application and online Advanced Data Access Tool used) | Image photo, larvae counts, habitat text descriptions, mosquito species records (from the app’s data fields including larvae, pupae, habitat type) | Expert validation implied through the Mückenatlas workflow; questionnaire respondents were people who had previously submitted *Anopheles plumbeus* specimens to the citizen-science project and received species-identification feedback from the project team, but this paper itself does not newly describe the specimen-identification procedure or report any validation-accuracy metric | Maximum Entropy modeling + VIF analysis + cross-validation | Not applicable (questionnaire / nuisance-management study rather than citizen-report-based ecological output analysis); the paper analyzed questionnaire responses on nuisance, breeding-site awareness, and control actions using logistic regression, not abundance, hotspot, temporal-trend, or risk outputs from uneven citizen-report intensity | Spatial predictions and environmental suitability maps | Participation bias and misidentification bias (clustering in Senegal, app misidentification, location bias) | Sampling bias; Data quality issue; Validation limitation; Recruitment/engagement challenge; Technology/access barrier | Implicit consent via voluntary app participation (no explicit ethics section, but participation is voluntary and public, data is open access) | GLOBE Observer uses account-based participation under NASA/GLOBE privacy rules; users register with an email address and country, public data are linked to a screen name rather than personal identifiers, email addresses are not publicly displayed, and photos are screened/blurred for faces or text before entering the database. | <https://github.com/eafreeman/aedes_globe> |
| [30](https://doi.org/10.1111/1365-2664.14417) | Validating a surveillance program of invasive mosquitoes based on citizen science in Hungary | László Zsolt Garamszegi | 2023 | Hungary | N/A | Urban/rural | descriptive; evaluation (study evaluates citizen science surveillance reliability) | 2949 reports (across 3 years as stated) | N/A | N/A | N/A | Data collection (citizens submit mosquito observations) | Open call (public invited via media campaign) | Surveillance (species identification, presence/absence tracking) | General mosquito monitoring (tracks Ae. albopictus, Ae. japonicus, Ae. koreicus) | Mobile apps; Web portal; smart phone (reports sent via Mosquito Alert app, email, website) | Image (photo) (public submits photos); mosquito species (species-level identification based on images) | Expert validation; citizen-submitted mosquito photos/specimens/reports; reports were validated by four experienced dipterologists who had to achieve 100% success on a 50-photo test before independent identification, with cross-validation and collective decisions in doubtful cases; Mosquito Alert app entries were validated through Ento Lab by three independent taxonomists under a regional coordinator; no single per-report accuracy percentage was reported, but citizen-science conclusions were later corroborated well against direct field sampling | Descriptive statistics; GLMs (Poisson); repeatability (GLMM); correlation analyses (statistical methods explicitly described: GLM, GLMM, correlation tests) | Explicit analytical adjustment for sampling effort; prevalence, presence/absence, and distribution-mapping outputs; the study used total reports per quadrat as a proxy for local sampling effort, modeled confirmed detections as a function of effort, estimated species-specific minimum sample-size thresholds to distinguish likely true absence from insufficient effort, and assessed repeatability/presence patterns only after controlling for study effort | Trend analysis; Risk-factor identification (they examine repeatability, spatial patterns, ecological predictors) | participation bias; data bias; generalizability (citizen science variation by region, sampling effort, photo quality) | Sampling bias; Data quality issue | Consent obtained from citizen scientists (implicit consent via data submission; privacy protected) | Project-level ethics were explicitly stated: the website/app/materials explained the study purpose and data-handling policy, and submitting observations implied consent for scientific use. For the Mosquito Alert component, the platform’s standard ethics include anonymous participation, instruction not to include personal information, linkage of reports to a random user ID, and open sharing/licensing of contributed data/images under project terms. | <https://doi.org/10.5061/dryad.np5hqbzzj> |
| [31](https://doi.org/10.1007/s10340-024-01841-7) | Identifying ecological factors mediating the spread of three invasive mosquito species: citizen science informed prediction | László Zsolt Garamszegi | 2025 | Hungary | N/A | Urban/rural | Descriptive analytical SDM study (evaluates predictors and generates predictive distribution maps using BRT models) | 4980 reports (uses full 5-year citizen science dataset for presence/pseudo-absence modeling) | N/A | N/A | N/A | Data collection; dataset annotation (citizens submit photos/specimens; scientists validate, analyze, and model distributions) | Open call (public invited via email, app, website, and nationwide media campaign) | Surveillance (species identification, mapping), vector control | General mosquito monitoring (invasive Aedes species, Ae. albopictus, Ae. japonicus, Ae. koreicus) | Mobile apps; email ; web portal (Mosquito Alert app + email submission + online platform; citizens submit geotagged observations) | Image (photo) + text metadata (photos, coordinates, submission mode; expert-validated) | Expert validation; citizen-submitted photos/specimens/reports collected through email, post, and the Mosquito Alert app; reports were processed by expert dipterologists who identified mosquito species, and only taxonomically validated records were used to define presence, while uncertain cases were handled conservatively as not confirmed for the focal species; no new per-report identification-accuracy metric was reported in this paper | Boosted Regression Trees (BRT), correlation screening, VIF, pseudo-absence weighting, logistic regression for validation (used to identify predictors and evaluate prediction accuracy) | Explicit analytical adjustment for sampling effort; probability-of-occurrence / species-distribution modelling outputs; quadrats with validated presence were given full weight, pseudo-absence quadrats were weighted according to underlying sample size, species-specific minimum sample-size thresholds from the prior surveillance-validation study were used to treat sufficiently sampled zero-detection quadrats as full-weight absences, and low-effort pseudo-absences were progressively downweighted | Risk-factor identification; Spatial predictions; risk models (continuous probability maps, species distribution predictions, ecological predictor importance) | Data bias, algorithm limitations, participation bias, generalizability (uneven sampling effort, spatial bias of citizen science, false negatives, class imbalance, scale mismatch between environment layers and mosquito movement, and limited generalizability for Ae. japonicus/Ae. koreicus) | Sampling bias; Spatial bias; Data quality issue; Representativeness/generalizability issue | Consent obtained from citizen scientists, privacy, data ownership | Mixed/project-level system. The paper reports that the citizen-science program followed explicit ethics guidance and protected participant anonymity; for the Mosquito Alert component, the platform requires agreement to its privacy policy/user agreement, supports anonymous participation, tells users not to include personal information, links reports to a random user ID, and allows sharing of contributed data/images under project and open-data licensing terms | <http://www.mosquitosurveillance.hu/> |
| [32](https://doi.org/10.4269/ajtmh.21-1127) | Community Participation in Habitat Management and Larviciding for the Control of Malaria Vectors in Southern Malawi | Steven Gowelo | 2023 | Malawi | N/A | Rural | Intervention, evaluation (community-led larval source management assessed over 14 months) | 502 survey participants + 26 LSM committees (502 KAP respondents; LSM committees coordinated implementation) | Adults ≥18 years (participants recruited from adult household members) | Mixed gender (60.8% female in KAP survey) | Predominantly local Malawian population (rural Malawian communities; no detailed ethnicity breakdown provided) | Data collection, monitoring interventions, engagement in meetings (LSM committees conducted mapping, draining/filling habitats, weekly larviciding, reporting) | Community orgs; village leadership (volunteers selected by village heads + HA-led recruitment) | Vector control; education (community engaged in larval source management + learning about malaria/vector biology) | Malaria (focus on Anopheles gambiae s.s., An. arabiensis, An. funestus) | field-based tools only (manual larviciding with Bti, area samplers, sprayers, ODK tablet for data capture) | larvae count, mosquito species (larval density counts, species-level sorting into Anopheles vs. culicines) | Not applicable; this study did not use a citizen-submitted mosquito observation/classification platform requiring validation of photos, specimens, or app-based records; instead, community members implemented habitat management and Bti larviciding, while an independent research team conducted standardized larval-density sampling and sorted collected larvae into anopheline versus culicine groups | Zero-inflated negative binomial models; chi-square tests; multivariable logistic regression (used to evaluate larval densities and factors influencing participation) | Not applicable (structured intervention and independent larval-surveillance study rather than opportunistic citizen reporting); outputs were based on standardized pre-/post-spray larval surveys and comparisons between LSM and non-LSM villages using zero-inflated negative binomial models, so participation/reporting-effort bias from ad hoc citizen observations was not the relevant analytical issue | Trend analysis, Risk-factor identification (evaluated changes in larval densities over time and factors influencing community participation; no hotspot maps or spatial predictions) | generalizability, participation bias (low larval densities due to prior LLIN scale-up; dry season; possible spillover between villages; operational constraints) | Recruitment/engagement challenge; Representativeness/generalizability issue | Consent obtained from citizen scientists (written informed consent from all community participants; privacy protected) | Not applicable as a platform-level ethics field. This was a locally organized community intervention, not a standing app/web platform with its own user-agreement/privacy/data-ownership system. Ethics were handled at the project level through ethics approval, community entry permission, and written informed consent for the KAP survey. | Not available |
| [33](https://doi.org/10.1111/tmi.12805) | Anopheles plumbeus (Diptera: Culicidae) in Germany: updated geographic distribution and public health impact of a nuisance and vector mosquito | Eva C. Heym | 2017 | Germany | N/A | Rural | Descriptive observational (study mapped spatial distribution, analyzed nuisance reports) | N/A | N/A | N/A | N/A | Data collection (citizens submitted mosquitoes to Mückenatlas—passive surveillance) | Open call (public invited to submit specimens in the Mückenatlas project :contentReference[oaicite:4]{index=4}) | Surveillance (species identification; presence/absence) (study used citizen-submitted specimens to detect presence and nuisance mass occurrence) | General mosquito monitoring (focus is Anopheles plumbeus distribution & nuisance, not disease-specific) | Web portal; smart phone (citizens submitted mosquitoes using *Mückenatlas* platform; implied web-based submission; digital photos possible) | Image (photo); mosquito species (citizens submitted physical specimens and likely photos; identification relied on morphology/genetics) | Expert validation; citizen-submitted adult mosquito specimens to the Mückenatlas project; citizens caught mosquitoes, froze them, and mailed them to the research institutions, where specimens were identified morphologically to species level using standard keys, and damaged specimens were identified genetically by CO1 barcoding; no formal identification-accuracy metric was reported | R statistical analysis; Kruskal-Wallis test (page 2 shows use of R, Kruskal–Wallis for comparisons) | Explicit analytical adjustment for reporting effort; distribution and nuisance-mapping outputs; because Mückenatlas submissions were considered biased by media attention, the study standardized Anopheles plumbeus submissions by the total number of mosquito submissions within each 50 × 50 km grid cell, excluded poorly sampled cells with ≤5 submissions, and used statistical comparisons to assess whether high species proportions were driven by uneven submission intensity | Spatial predictions; Trend analysis (study mapped national distribution, standardized submissions, analyzed spatial trends) | participation bias; generalizability (citizen submissions uneven across regions, media attention bias noted; possible underrepresentation) | Sampling bias; Data quality issue | Consent obtained from citizen scientists (participants voluntarily submitted specimens; privacy maintained; no personal data reported) | Mückenatlas uses an explicit consent/data-protection framework: participants submit a form and consent to data processing; personal data are handled confidentially, research partners receive mosquito and location information in anonymized form, contributors may appear on the collector map only if they opt in, can use a pseudonym, and can withdraw consent later. | Not available |
| [34](https://doi.org/10.3389/fpubh.2017.00278) | The Nuisance Mosquito Anopheles plumbeus (Stephens, 1828) in Germany-A Questionnaire Survey May Help Support Surveillance and Control | Eva C. Heym | 2017 | Germany | N/A | Rural (community-based) | Descriptive survey study (questionnaire-based descriptive analysis) | 118 participants | Adults | Male and female (with more males responding) | N/A | Data collection; engagement in research, communication (Level of Citizen Involvement, citizens submitted mosquitoes and completed questionnaires; engaged with project team) | Open call (participants were people who submitted samples to the Mueckenatlas project) | Surveillance (species presence, nuisance assessment, breeding site identification) (Purpose: citizens helped identify nuisance, sources, and environmental context) | General mosquito monitoring (focus on Anopheles plumbeus as nuisance and potential vector) | Web portal; citizen submission tool (citizens used Mueckenatlas submission portal; no mobile app used) | Text; mosquito specimens (questionnaire responses & submitted mosquitoes) | Expert validation implied through the Mückenatlas workflow; questionnaire respondents were people who had previously submitted *Anopheles plumbeus* specimens to the citizen-science project and received species-identification feedback from the project team, but this paper itself does not newly describe the specimen-identification procedure or report any validation-accuracy metric | Logistic regression, Descriptive statistics, chi-square tests, | Not applicable (questionnaire / nuisance-management study rather than citizen-report-based ecological output analysis); the paper analyzed questionnaire responses on nuisance, breeding-site awareness, and control actions using logistic regression, not abundance, hotspot, temporal-trend, or risk outputs from uneven citizen-report intensity | Risk-factor identification (logistic regression identified environmental predictors of nuisance) | Data bias, participation bias, misidentification of breeding sites, generalizability limitations (survey only included people who submitted samples; uneven geographic representation; limited identification accuracy) | Sampling bias; Data quality issue; Validation limitation | Consent obtained; anonymity maintained (written informed consent; anonymous evaluation) | Mückenatlas uses an explicit consent/data-protection framework: participants submit a consent form, personal data are processed confidentially, mosquito and location data are shared with research partners in anonymized form, contributors can appear on the collector map only if they opt in, may use a pseudonym, and can withdraw consent later. | <http://www.frontiersin.org/article/10.3389/fpubh.2017.00278/full#supplementary-material> |
| [35](https://doi.org/10.1186/s13071-021-05077-7) | The invasive Korean bush mosquito Aedes koreicus (Diptera: Culicidae) in Germany as of 2020 | Nicolas Hohmeister | 2021 | Germany | N/A | Urban (study sites across Wiesbaden, Munich) | Descriptive, surveillance evaluation (field sampling) | N/A | N/A | N/A | N/A | Data collection (citizens submitted mosquitoes enabling detection) | Volunteers (citizens submitted via Mueckenatlas) | Surveillance (species identification, detection of invasive species) | General mosquito monitoring (focus on Ae. koreicus distribution) | Web portal, smart phone (Mueckenatlas online submission system) | Image (photo), mosquito species (specimens + ID data) | Expert validation with morphological and genetic confirmation; citizen-submitted adult bush mosquitoes and field-collected larvae/adults; specimens suspected to be *Aedes koreicus* or showing ambiguous characters were examined morphologically and then confirmed genetically by PCR/sequencing (CO1 and/or ND4 markers), with archived “*Ae. japonicus*” material and recent Mueckenatlas submissions re-checked particularly carefully; no formal identification-accuracy percentage reported | Phylogenetic analysis, descriptive summaries (ND4, CO1 markers) | Not applicable (presence/establishment surveillance and targeted re-examination study rather than citizen-report-based quantitative output analysis); the study used cemetery inspections, archived specimen re-analysis, and Mueckenatlas detections to document occurrence, establishment, and limited spread of *Ae. koreicus*, but it did not estimate abundance, hotspots, temporal trends, or risk outputs requiring explicit correction for reporting/sampling effort | Trend analysis, Spatial predictions (spread assessment over time) | data bias, algorithm limitations, participation bias, generalizability (sampling limits + ND4 resolution issues) | Validation limitation; Representativeness/generalizability issue; Data quality issue | Not applicable, privacy ensured (no human subjects; anonymized submissions) | Mückenatlas uses an explicit consent/data-protection framework: participants submit with consent, personal data are handled confidentially, mosquito and location data are shared with research partners in anonymized form, contributors appear on the collector map only if they opt in, may use a pseudonym, and can withdraw consent later. | Included in the manuscript |
| [36](https://doi.org/10.1007/s10393-025-01711-3) | Citizen Science Detection and Characterization of Mosquito-Borne Viruses | Lucas Hollett | 2025 | Canada; France | Newfoundland and Labrador | Rural/remote & community-based (citizen samples came from remote rural areas and residences) | Descriptive surveillance study (design focuses on detecting species & viruses through citizen sampling) | 72 recruited (2018) + 182 recruited (2019) | N/A | N/A | N/A | Data collection & sample submission (citizens collected mosquitoes and submitted samples) | Open call; via schools, community organizations, social media (stated outreach + partnerships) | Surveillance of mosquito species & virus detection (goal was distribution + virus identification) | General mosquito monitoring; California serogroup viruses (SHV, JCV-like) | Manual aspirators, PCR, sequencing, social media communication | Physical specimens, photos, location info | Expert validation with molecular confirmation; citizen-submitted mosquito specimens collected with aspirators; samples were identified under a microscope by a trained and certified mosquito identifier, and PCR plus Sanger sequencing were used when species confirmation was needed; virus-positive pools were further characterized by PCR and sequencing | Descriptive summaries & molecular phylogenetics | Presence/diversity and virus-detection surveillance; no explicit analytical correction for participation effort reported; the study mapped collection sites and described species occurrence and virus detections, while uneven response rates, sample-quality issues, non-uniform seasonal collection, and the focus on human-attracted mosquitoes were discussed as limitations rather than corrected analytically | Geographic viral patterns; phylogenetic trees | Sample quality issues, uneven sampling, COVID-19 disruption, species bias | Recruitment/engagement challenge; Data quality issue; Sampling bias; Representativeness/generalizability issue | Not applicable | Not applicable as a platform-level ethics field. This was a project-specific citizen-science system, not a standing app/web platform with its own standard user-agreement, privacy, or data-ownership framework clearly described in the paper. | Not available |
| [37](https://doi.org/10.13057/biodiv/d260813) | Community-led application of Bacillus thuringiensis var. israelensis (Bti) effectively reduces malaria vector densities in The Gambia | BABUCARR JASSEY | 2025 | Gambia | N/A | Urban/rural | intervention, evaluation (The study tests the effectiveness of community-led vs expert-supervised Bti applications) | N/A | N/A | N/A | N/A | Data collection, monitoring interventions, engagement in research (Community members identified breeding sites, applied Bti, and monitored mosquito populations) | community volunteers, local leaders, health authorities (Recruitment described as selection of village health workers, volunteers, and farmers) | Surveillance + vector control (Purpose was to identify breeding sites and apply Bti) | Malaria (Bti targets Anopheles vectors) | CDC light traps, dipping tools, water-dispersible granules, sprayers | larvae count, pupae count, adult mosquito count, breeding sites, mosquito species | Not applicable; this study did not use a citizen-submitted mosquito observation, image, or specimen-classification platform requiring validation of citizen-generated records. Instead, community members were trained to identify breeding sites and apply Bti, while entomological outcomes were measured through standardized larval sampling and CDC light-trap adult collections analyzed within the study framework | GLMM (Generalized Linear Mixed Models used for analysis) | Not applicable (structured intervention and standardized entomological monitoring rather than opportunistic citizen reporting); larval, pupal, and adult Anopheles outcomes were based on repeated biweekly surveys, CDC light traps, and generalized linear mixed models / negative binomial regression comparing intervention and control arms, so participation/reporting-effort bias from ad hoc citizen observations was not the relevant analytical issue | trend analysis, risk-factor identification, impact evaluation (Findings include temporal population trends and intervention effectiveness) | Participation bias, environmental variability, uneven application, generalizability | Recruitment/engagement challenge | Consent and community approval | Not applicable as a platform-level ethics field. This was a field intervention program, not a standing digital platform with its own user-agreement, privacy, or data-ownership framework described in the paper. | Not available |
| [38](https://doi.org/10.46471/gigabyte.54) | Mosquito alert: leveraging citizen science to create a GBIF mosquito occurrence dataset | Živko Južnič-Zonta | 2022 | Spain & Europe (because most data come from Spain, with later expansion to multiple European countries) | N/A | Urban | Descriptive, surveillance evaluation | N/A | N/A | N/A | N/A | Data collection; annotating dataset; engagement in research (citizens submit photos, geolocations, and metadata) | Open call (anyone worldwide can participate using the Mosquito Alert app) | Surveillance (species identification, presence/absence mapping) + early warning for vector control (dataset supports MBD risk assessment) | General mosquito monitoring (Dengue, Zika, chikungunya, West Nile (because species include Ae. albopictus, Ae. aegypti, Ae. japonicus, Ae. koreicus, Cx. Pipiens, vectors of these diseases) | Mobile app; Web portal (mosquito alert) (EntoLab); GPS (submissions are geolocated and validated by experts online) | Images (photos); text; mosquito species; geolocation (citizens submit photos, comments, and location coordinates) | Expert validation (multi-expert consensus); citizen-submitted geolocated adult-mosquito photographs; each report was independently reviewed by three entomologists through the digital EntoLab platform, the final species label was assigned by majority vote with certainty classified as probable or confirmed, and flagged/conflicting reports were reviewed by a senior “super expert”; no formal validation-accuracy percentage was reported | Not applicable (data release; no statistical modeling performed in this article) | Platform-level effort-bias correction described; dataset / occurrence-surveillance paper rather than study-specific bias-sensitive ecological analysis; the app collects anonymous information on the geographic distribution of participants to correct sampling-effort biases, but this paper mainly describes the occurrence dataset and validation system rather than applying a study-specific effort-adjustment model to abundance, hotspot, or temporal-trend outputs | Risk models; spatial predictions; trend analysis (the dataset is intended for modeling vector exposure, early-warning systems, and ML detection) | Participation bias; sampling bias; algorithm limitations; generalizability limits (author notes that submissions vary by region, season, and app engagement) | Sampling bias; Validation limitation; Data quality issue | Consent obtained via in-app User Agreement; anonymous participation; privacy protection measures (participants must accept the agreement before using the app) | Mosquito Alert requires acceptance of its user agreement and privacy policy, supports anonymous participation, asks users not to include personal information, links reports to a randomly generated user ID, and allows submitted images/data to be shared under project licensing and open-data terms. The paper also states that participation is anonymous and that publication consent is given at registration. | <https://www.gbif.org/dataset/> |
| [39](https://doi.org/10.1029/2021gh000436) | GLOBE Mosquito Habitat Mapper Citizen Science Data 2017-2020 | Russanne Low | 2021 | Global (data from 73 countries) | N/A | Urban/rural | Descriptive observational | 24,983 total observations; 5,138 complete records (reported in dataset summary) | 13+ (app allows users aged 13 and up) | N/A | N/A | Data collection; monitoring interventions (citizens document habitats and perform source-reduction mitigation actions) | Volunteers; community orgs (participation occurs through voluntary public use and GLOBE training events) | Surveillance; education; vector control (users document larval habitats, learn identification, and eliminate breeding sites) | General mosquito monitoring (Aedes, Culex, Anopheles larvae observed) | Mobile apps; smart phone (GLOBE Observer app on iOS/Android used for all data entry) | Image (photo); larva count; breeding sites (photos of habitats/larvae, numerical larval counts, habitat descriptions) | Participant self-identification with voucher-photo support; mosquito larval habitats and larvae were documented through the app, and users could identify larvae to genus using an in-app pictorial dichotomous key; submitted whole-body and close-up photos served as voucher specimens for validating identifications, but this paper did not describe a systematic expert-validation workflow for all records or report a formal identification-accuracy metric | Descriptive statistics; data cleaning algorithms; geolocation filtering (Python scripts used to clean, filter, and summarize data) | Data-quality filtering and bias explicitly examined, but not formally corrected with an effort-adjustment model; opportunistically collected larval habitat / genus-report dataset; the authors removed records with insufficient or anomalous geolocation, removed suspected duplicate training-event records after visual inspection, and explicitly discussed spatial-temporal opportunistic sampling bias as a limitation that constrains broad inference, especially where geospatial density is low | Trend analysis; risk-factor identification (data used to examine spatial patterns and support disease-risk modeling) | Data bias; participation bias; generalizability (opportunistic sampling, uneven spatial density, uneven reporting noted as limitations) | Sampling bias; Data quality issue; Validation limitation; Technology/access barrier | Consent obtained from citizen scientists; privacy; data ownership (participants accept GLOBE User Agreement for anonymous data use) | GLOBE Observer uses account-based participation under NASA/GLOBE privacy rules; users register with an email address and country, public data are linked to a screen name rather than personal identifiers, email addresses are not publicly displayed, and photos are screened or blurred for faces/text before entering the database. | https://www.globe.gov/globe-data (open public dataset link provided) |
| [40](https://doi.org/10.3390/insects13070624) | Building International Capacity for Citizen Scientist Engagement in Mosquito Surveillance and Mitigation: The GLOBE Program's GLOBE Observer Mosquito Habitat Mapper | Russanne Low | 2022 | USA | N/A | Urban/rural | Descriptive (paper describes the tool, methods, outreach, education, deployments) | N/A | N/A | N/A | N/A | Data collection and monitoring interventions (citizens document larval habitats, count larvae, identify larvae, and perform source reduction) | Open call (participation through app, outreach campaigns, schools, libraries, community groups) | Surveillance and education (citizens identify habitats, larvae, mitigate breeding sites; educational outreach central to program) | General mosquito monitoring (focus on Aedes, Culex, Anopheles, invasive species; not tied to one disease) (tool covers habitats & larvae across taxa, not disease-specific) | Mobile apps, Web portal, GPS, smart phone (GLOBE Observer app; GPS-based geolocation; visualization & ADAT portals) | Image (photo), text, larva count, mosquito species (voucher photos, counts, habitat descriptions, optional ID) (Step 1–3 documentation) | Mixed validation; participant self-identification with voucher-photo support, plus expert manual validation for selected datasets/use cases; citizen scientists used an in-app pictorial dichotomous key to identify larvae, uploaded voucher photos, and expert optical validation was performed for subsets of larval photos, while AI/computer-vision validation was under development; in one expert-validated subset, accuracy of attempted citizen-scientist larval identifications varied by country (Benin 55%, Kenya 100%, Senegal 34%, Madagascar 100%) | No statistical analysis (paper does not report statistical modeling; it focuses on system design, validation, outreach) (only descriptive summaries presented) | Bias explicitly discussed; design-based mitigation/support for systematic sampling, but no formal study-specific effort-correction model applied; the paper states that Mosquito Habitat Mapper data are often opportunistically collected and spatially biased, especially around where people live, but also notes that the app can support systematic sampling through project design, team-based data collection, and geofencing for location-specific requests | Trend analysis & Risk-factor identification (used to study habitat types, species occurrence, climate/land cover associations) | Data bias, participation bias, generalizability (paper explicitly discusses sampling bias, user errors, opportunistic sampling, device differences affecting ID) (Sections 2.5–2.9) | Sampling bias; Data quality issue; Validation limitation; Technology/access barrier; Recruitment/engagement challenge | Consent obtained, privacy, data ownership (app collects anonymized data; open data but no personal identifiers; implicit consent via participation) (based on GLOBE open-data & citizen scientist protection principles) | GLOBE Observer uses account-based participation under NASA/GLOBE privacy rules; users register with an email address and country, public data are linked to a screen name rather than personal identifiers, email addresses are not publicly displayed, and photos are screened or blurred for faces/text before entering the database. | <https://www.globe.gov/globe-data> |
| [41](https://doi.org/10.2196/publichealth.7376) | Lessons From the Implementation of Mo-Buzz, a Mobile Pandemic Surveillance System for Dengue | May Oo Lwin | 2017 | Sri Lanka | N/A | Urban | Participatory surveillance system / descriptive evaluation (study describes development, implementation, and evaluation), | PHIs ~50; Public 513 baseline; 80 pilot users (all sample sizes reported), | Adults (participants were adult PHIs and adult community members) | N/A | N/A | Data collection, monitoring, civic engagement (PHIs digitize data; public reports symptoms and breeding sites), | Community orgs; baseline surveys + institutional recruitment (recruited via CMC, Mobitel, university networks), | Surveillance (symptoms, breeding sites), education, outbreak awareness (defined purpose), | Dengue | Mobile app, GPS, smartphone camera (Mo-Buzz is a smartphone-based system) | Image (photo), text, symptom data, breeding-site photos | Not stated / not described; the public version of Mo-Buzz allowed users to report dengue symptoms and upload geotagged pictures of potential mosquito-breeding sites to health authorities, but the paper did not describe a formal validation or classification workflow for these public submissions, who reviewed them, or any accuracy assessment | Descriptive statistics; qualitative thematic analysis (NVivo-coded interviews + uptake metrics), | Not applicable (implementation and uptake study rather than citizen-report-based ecological output analysis); the paper evaluated system development, usability, PHI adoption, and public receptivity, while hotspot mapping was described as a platform feature based on archival and current dengue-related data rather than an analysis of opportunistic citizen observations requiring correction for participation/reporting effort | Hotspot maps, outbreak prediction, real-time surveillance | Data bias, algorithm limitations, participation bias, generalizability | Technology/access barrier; Recruitment/engagement challenge; Data quality issue | Consent obtained from citizen scientists, privacy | No clear public platform-level ethics framework was reported. Ethics appear to be mainly project-level, as the paper states that the research was approved by the institutional review board of Nanyang Technological University. | <https://publichealth.jmir.org/2017/4/e65/> |
| [42](https://doi.org/10.1186/s12936-025-05564-7) | Fighting against malaria is everyone's concern: a randomized control trial assessing the role of incentives for encouraging local communities to record and upload mosquito sounds using the MozzWear application | Winifrida P. Mponzi | 2025 | Tanzania | N/A | Rural | Randomized controlled trial | 144 participants | Adults 18–69 years | Male & Female (49% male, 51% female) | N/A | Data collection (citizens recorded and uploaded mosquito sound data using phones) | Community meetings (participants recruited during local village meetings) (leaders invited researchers; volunteers selected during meetings) | Surveillance (recording flight tones; identifying mosquitoes; contributing to malaria control) | Malaria | Smartphone + Mobile app + Sensor (MozzWear app + HumBug acoustic sensor + smartphones) | Audio recordings of mosquito flight tones (participants recorded mosquito sounds at night) | Automated acoustic detection/classification platform; community participants used HumBug smartphones running the MozzWear app to record host-seeking mosquito flight tones overnight, and the system is described as passively detecting and identifying mosquitoes from their sounds; however, this paper did not assess validation accuracy, species-classification performance, or any separate human verification workflow | Poisson GLM + descriptive statistics (used to compare number of successful uploads; R statistical software)—(data analysis section) | Not applicable (participation/incentives study rather than ecological surveillance-output analysis); the main outcome was the number of nights with successful mosquito-sound recording and upload, analysed across intervention/control groups and demographic variables, rather than abundance, hotspot, temporal-trend, or risk outputs requiring correction for citizen-reporting effort | Trend analysis (uploads across weeks; demographic comparisons) (trained on weekly trends and demographic patterns; no spatial prediction) | Participation bias + generalizability (differences in motivation; limitations with phone misuse, device damage, connectivity issues) | Technology/access barrier; Data quality issue; Representativeness/generalizability issue | Consent obtained; privacy considerations (written individual informed consent; community permission; IRB approvals) | HumBug/MozzWear has a privacy-focused project policy: the app records environmental sound, uses real-time voice-activity detection, automatically deletes clips containing human voice, and deployment areas should be clearly signposted; the HumBug website also follows the University of Oxford privacy policy. | Available upon request |
| [43](https://doi.org/10.7554/elife.27854) | Using mobile phones as acoustic sensors for high-throughput mosquito surveillance | Haripriya Mukundarajan | 2017 | USA & Madagascar | California; Ranomafana (Ifanadiana District) | Urban | Pilot; Proof-of-concept field evaluation (feasibility demonstrated via small-scale field trials) | ~25 volunteers total (15 in CA, 10 in Madagascar) (volunteers participated in both field sites) | N/A | N/A | N/A | Data collection (citizens captured acoustic mosquito recordings only) | Volunteers; recruited for field trials (participants engaged as field volunteers) | Surveillance (species identification and presence/absence mapping) | General mosquito monitoring (species relevant to malaria, dengue, Zika, and other vector-borne diseases) | Smart phone; GPS; Mobile apps (audio recording apps + built-in phone sensors) | Audio recordings; mosquito species (acoustic signatures captured for species identification) | Automated acoustic classification with independent technical and field validation; mosquito wingbeat sounds recorded by mobile phones were classified using species-specific wingbeat-frequency distributions and a maximum likelihood estimation (MLE) framework, with audio frequencies first validated against synchronized high-speed video showing an exact time-aligned match within a small computational error and high distributional overlap, and field identities further checked by capturing corresponding specimens for morphological identification; reported performance included improvement in average classification accuracy from about 35% to 65% when location metadata were added, and about two-thirds correct classification for wild *Aedes sierrensis* recordings (54/82) | MLE, STFT, Bhattacharya Coefficient, Jensen-Shannon Divergence (methods used for classification) | No formal analytical correction for participation/reporting effort; structured volunteer-based spatio-temporal mapping and species-screening outputs; minimally trained users pooled mobile-phone recordings to map human-mosquito encounters and local mosquito distributions over defined field periods and locations, with comparison to CDC light traps and site-specific reference databases, but the study did not model observation/reporting effort explicitly; the Discussion also notes that the method is inherently biased toward anthropophilic species because collection depends on human involvement | Spatial predictions; Trend analysis; Risk-factor identification (spatio-temporal mapping of mosquito activity) | Data bias, algorithm limitations, participation bias, generalizability (bias toward anthropophilic species; noise; phone variability) | Sampling bias; Data quality issue; Validation limitation; Technology/access barrier; Representativeness/generalizability issue | onsent obtained from citizen scientists, privacy, data ownership (participants anonymous; identifiers removed) | Not applicable as a platform-level ethics field. This was a project-specific mobile phone recording approach, and the paper does not report a standing public user-agreement, privacy, or data-ownership framework for a named platform. | <https://doi.org/10.5061/dryad.98d7s> |
| [44](https://doi.org/10.1016/j.njas.2018.07.005) | A citizen science approach for malaria mosquito surveillance and control in Rwanda | Marilyn Milumbu Murindahabi | 2018 | Rwanda | N/A | Rural | Descriptive, conceptual framework study (justification: paper describes surveillance system, determinants, and a proposed citizen science approach rather than an intervention) | N/A | N/A | N/A | N/A | Data collection; engagement in research (citizens collect mosquito nuisance data and participate in monitoring; described as observation, reporting, and involvement with CHWs) | Community orgs; via CHWs (community members report through Community Health Workers; no open-call volunteer sampling) | Surveillance; vector control ; community education (aim is spatio-temporal mosquito monitoring, nuisance reporting, and malaria control awareness) | Malaria | Web portal; Mobile apps; GPS; Smartphones (the approach proposes SMS, web tools, and future mobile apps for monitoring) | Text, mosquito species, breeding sites (citizens report mosquito nuisance, habitats, and specimens) | Not applicable / not described; this paper presents a conceptual citizen-science framework for malaria mosquito surveillance in Rwanda rather than an implemented surveillance platform with submitted images, specimens, or records undergoing a defined validation workflow; it proposes community reporting of mosquito nuisance and other entomological information through paper-based forms and later digital/web/mobile tools, but does not describe who would validate observations or any identification-accuracy assessment | No statistical analysis (justification: conceptual framework paper; no data analysis reported) | Not applicable (conceptual framework paper rather than citizen-data surveillance-output analysis); the paper argues that citizen science could improve coverage, especially in remote areas currently excluded from mosquito monitoring, but it does not analyse citizen-generated data or apply any analytical correction for reporting/sampling effort | Risk models (potential for linking entomological data with climate data), Spatial predictions (integration with GIS and ENACTS malaria map room) | Participation bias, generalizability, data bias ( issues such as lack of infrastructure, low participation, inconsistent data, and remote-area limitations) | Spatial bias; Technology/access barrier; Recruitment/engagement challenge | Not applicable | Not applicable as a platform-level ethics field. This paper mainly describes a proposed framework, and it does not report a standing public user-agreement, privacy, or data-ownership policy for an existing named platform. | Not available |
| [45](https://doi.org/10.1186/s12936-021-03989-4) | Citizen science for monitoring the spatial and temporal dynamics of malaria vectors in relation to environmental risk factors in Ruhuha, Rwanda | Marilyn Milumbu Murindahabi | 2021 | Rwanda | N/A | Rural | descriptive, observational (long-term monitoring without intervention) | 112 volunteers | median 42, range 24–68 | 51% male, 49% female | N/A | Data collection (volunteers collected mosquitoes, nuisance, malaria case data) | volunteers (recruited through workshops & interest-based sign-up) | surveillance (species identification, presence/absence, trap monitoring) (core activity of program) | Malaria | sensor (CO₂-baited trap); paper forms (reporting tool) | mosquito species, mosquito counts, text nuisance scores, malaria case reports | Expert/laboratory validation of citizen-collected physical specimens; volunteers collected mosquitoes monthly using standardized handmade carbon-dioxide-baited traps, after which researchers morphologically identified specimens with taxonomic keys, tested female *Anopheles* for *Plasmodium falciparum* circumsporozoite protein by ELISA, and identified a subset of *An. gambiae* s.l. by PCR to sibling-species level; no formal citizen-versus-expert accuracy metric was reported | Spearman correlation, Pearson correlation, multiple regression, PCA, negative binomial GLM | Structured standardized citizen-assisted sampling; no explicit analytical correction for participation/reporting effort; abundance, hotspot, correlation, and environmental-risk outputs were based on fixed monthly household surveillance using two standardized traps per household plus repeated reporting procedures, and the authors additionally tested models with and without village to assess spatial clustering, but they did not include a separate effort-adjustment, reporting-intensity, or detection-probability correction term | hotspot maps, spatial predictions, risk-factor identification (IDW maps; elevation & river distance predictors) | participation bias, generalizability (uneven distribution; low trap efficiency) | Sampling bias; Data quality issue | consent obtained from citizen scientists (IRB approval & informed consent) | Not applicable as a platform-level ethics field. This was a project-specific citizen-science system, not a standing app/web platform with its own standard user-agreement, privacy, or data-ownership framework clearly described in the paper. | https://doi.org/10.17026/dns-xhq-jtfs |
| [46](https://doi.org/10.1371/journal.pone.0156388) | Crowdsourcing Vector Surveillance: Using Community Knowledge and Experiences to Predict Densities and Distribution of Outdoor-Biting Mosquitoes in Rural Tanzania | StephenPeterMwangungulu | 2016 | Tanzania | N/A | Rural | Descriptive; participatory GIS evaluation (study tests and validates a crowdsourcing/vector surveillance approach, not an intervention trial) | 720 | Adults + schoolchildren, ages 11–18+ | 50% male and 50% female | N/A | Data collection; engagement in research (Participants ranked grids, participated in mapping sessions, and provided experiential knowledge, aligns with data collection and engagement) | Community orgs; volunteers (Participants recruited with help from village leaders, teachers, and community volunteers) | Surveillance (Predicting mosquito abundance hotspots through community ranking) | Malaria (Primary vector focus was Anopheles mosquitoes) | GPS; paper maps (study used handheld GPS receivers, printed maps, grid maps; no mobile apps or sensors used) | Text; mosquito species (Participants provided grid rankings, textual data; entomological traps provided species-level mosquito counts) | Independent entomological validation of community-ranked mosquito-density maps; community members did not classify mosquito specimens or images, but instead ranked village grids by expected outdoor mosquito density, and these predictions were then verified using odor-baited outdoor traps placed in areas classified as high, medium, and low density; trapped mosquitoes were morphologically identified and sorted by taxa, and the study showed that entomological catches consistently matched the community-defined density classes across villages and months | GLM; descriptive statistics | Structured community-ranking design with independent validation; no explicit analytical correction for participation/reporting effort; community members ranked all village grids on a 1–5 scale, the rankings were interpolated by IDW and reclassified into high/medium/low density categories, and mosquito catches were then modelled as a function of community-perceived density category and month using GLMs, but the study did not include a separate reporting-intensity, detection-probability, or sampling-effort correction term | Trend analysis; Spatial predictions; Risk-factor identification (community-generated density surfaces, IDW interpolation, village-wide spatial predictions, temporal trends over 12 months) | Participation bias; data bias; generalizability (reliance on local knowledge, variable accuracy, limitations of crowdsourcing, need for validation in more sites) | Validation limitation; Data quality issue; Spatial bias | Consent obtained (Detailed informed consent process described in ethics section; privacy maintained; approvals from institutional review boards) | Not applicable as a platform-level ethics field. This was a project-specific community participatory system, not a standing app/web platform with its own standard user-agreement, privacy, or data-ownership framework. Ethics were handled at the project level through IRB approval, written informed consent, and guardian/teacher consent for minors. | All relevant data are within the paper |
| [47](https://doi.org/10.1038/s41598-024-73416-6) | Assessing and correcting neighborhood socioeconomic spatial sampling biases in citizen science mosquito data collection | Álvaro Padilla-Pozo | 2024 | Spain | N/A | Urban | evaluation (evaluates citizen science sampling bias and surveillance performance) | N/A | N/A | N/A | N/A | Data collection, data analysis (citizens submit mosquito reports/photos; data used in modeling) | open call (any citizen can use Mosquito Alert; uN/Aestricted participation) | Surveillance (species identification, presence/absence, nuisance reporting) | General mosquito monitoring (focus on Ae. albopictus but not tied to a single disease) | Mobile apps, Sensor, GPS, Web portal (Mosquito Alert platform components) | Image (photo), text, mosquito species (photos, classification, presence data) | Expert validation of citizen-submitted photo reports, with platform-level AI support described; the Mosquito Alert system is described as combining expert validation and artificial intelligence, and all adult reports with photographs are reviewed in the Digital Entolab system by entomologists who score the probability that the report represents one of the target vector species; for this study, the mosquito-presence analyses specifically used the subset of expert-validated *Aedes albopictus* reports | Bayesian logistic regression, ICAR spatial models, zero-inflated Poisson, spatiotemporal modeling | Explicit analytical correction for socioeconomic spatial sampling bias and participation effort; high-resolution *Ae. albopictus* presence/risk modelling; the study first modeled reporting probability using Mosquito Alert adult/bite reports around ASPB-confirmed active catch basin drains, incorporated Mosquito Alert anonymized background-tracking sampling-effort estimates, then used the predicted sampling probabilities to weight pseudo-absences and included the log of predicted sampling effort as an offset in the mosquito vector model to correct citizen-science bias | Risk models, Spatial predictions (Ae. albopictus probability surfaces, bias-corrected maps) | data bias, participation bias, socioeconomic sampling bias, limited generalizability | Sampling bias; Validation limitation; Data quality issue | privacy (uses anonymized geolocation; no demographic data collected to protect participants) | Mosquito Alert requires acceptance of its user agreement and privacy policy, supports anonymous participation, asks users not to include personal information, links reports to a randomly generated user ID, and allows submitted images/data to be shared under project licensing and open-data terms. This paper also states that all participants provide informed consent before registering or submitting data. | https://zenodo.org/doi/10.5281/zenodo.10684356 |
| [48](https://doi.org/10.1038/s41467-017-00914-9) | Citizen science provides a reliable and scalable tool to track disease-carrying mosquitoes | John R.B. Palmer | 2017 | Spain | N/A | Urban | Descriptive; evaluation study (compares citizen science vs ovitrap surveillance methods) | >38,400 registered participants; 4,767 reliable reports analyzed (numbers reported in methods section) | N/A | N/A | N/A | Data collection; annotating dataset (citizens report mosquito sightings and provide photos; experts validate) | Open call; via mobile app volunteers (participants join by downloading and using Mosquito Alert) | Surveillance (species identification, presence/absence mapping, early warning detection) | Aedes albopictus | Mobile app, smartphone sensors, GPS (Mosquito Alert platform described in methods) | Images, text, geolocation, species presence reports (photo + taxonomic survey + GPS) | Expert validation with participant-supported classification; Mosquito Alert citizen-submitted adult tiger-mosquito reports; reports with photographs were reviewed by a team of entomologists and classified into categories such as “confirmed Tiger,” “probably Tiger,” “unclassifiable,” “probably not Tiger,” and “surely not Tiger”; for early-warning analyses the study used only expert-validated “confirmed” or “probable” reports, while for the human–mosquito encounter analysis it also included reports without clear photos when participants’ taxonomic survey responses supported reliability; surveillance performance against ovitrap data suggested high specificity (about 97%) for early-warning detections, though no per-image identification-accuracy percentage was reported | Bayesian multilevel logistic regression, ROC analysis, WAIC model comparison | Explicit analytical correction for sampling effort; early-warning and human–mosquito encounter probability outputs; the study modeled participant reporting propensity as a function of participation time and intrinsic motivation, used anonymous background location sampling to estimate participant geographic distribution, calculated sampling effort from participant-sightings weighted by reporting propensity, and then estimated biweekly alert probabilities conditional on sampling effort as a proxy for human–mosquito encounter probability | Spatial predictions, early warning maps, encounter probability models | Participation bias, sampling bias, uneven spatial representation | Sampling bias; Validation limitation; Recruitment/engagement challenge; Spatial bias | Consent; privacy protections (location rounding) | Mosquito Alert requires acceptance of its user agreement and privacy policy, supports anonymous participation, asks users not to include personal information, links records to protected participant identifiers rather than exact public identities, and shares data/images under project and open-data licensing terms (including CC0 for part of the database). | <https://doi.org/10.5281/zenodo.6465316> |
| [49](https://doi.org/10.1038/s41598-021-83657-4) | Deep learning identification for citizen science surveillance of tiger mosquitoes | Balint Armin Pataki | 2021 | Spain | N/A | Urban | descriptive (describes model training, dataset, citizen science input) | 7,686 images / 7,168 reports (citizen-submitted dataset size) | N/A | N/A | N/A | Data collection (citizens upload photos only) | Open call (any user can use the Mosquito Alert app) | Surveillance (species identification via citizen photos) | Aedes albopictus (dengue, Zika, chikungunya | Mobile app, GPS, smart phone (Mosquito Alert smartphone system) | Image (photo) (citizen-submitted mosquito photos) | Expert validation with AI-assisted image classification development; Mosquito Alert citizen-submitted mosquito photos; uploaded images were classified by entomology experts, with each image validated by one expert into categories (*Ae. albopictus*, *Ae. aegypti*, other species, or cannot tell) and marked as probable or confirmed, and this curated expert-validated image set was then used to train a ResNet50 deep-learning classifier for tiger-mosquito identification; reported model performance was very high (ROC AUC about 0.96, and about 98% accuracy for roughly 80% of images when uncertain cases were deferred to human review), though no inter-expert agreement metric was reported | ROC AUC, accuracy, sensitivity, specificity, confusion matrix, bootstrapping (used to evaluate CNN model) | Not applicable for this study’s main analysis; image-classification/validation study rather than ecological surveillance-output modelling; the paper notes that Mosquito Alert as a platform estimates participants’ sampling effort from background geo-positioning to support mosquito-risk inference, but this article itself focused on expert-labeled image classification and deep-learning performance, and did not apply a study-specific analytical correction for participation/reporting effort, detection probability, or spatial sampling bias | Risk-factor identification (model identifies Aedes presence for surveillance) | data bias, algorithm limitations, generalizability (dataset imbalance and image-quality issues) | Data quality issue; Validation limitation; Sampling bias | privacy (citizen-submitted images & geolocation; no personal identifiers) | Mosquito Alert requires acceptance of its user agreement and privacy policy, supports anonymous participation, asks users not to include personal information, links records to protected/random participant identifiers rather than exact public identities, and shares submitted images/data under project and open-data licensing terms. | http://www.mosquitoalert.com/en/mosquito-images-data-base/ |
| [50](https://doi.org/10.3390/insects12050374) | Buzzing Homes: Using Citizen Science Data to Explore the Effects of Urbanization on Indoor Mosquito Communities | Nadja Pernat | 2021 | Germany | N/A | urban/rural | descriptive (observational analysis of citizen-submitted samples) | 16,933 submissions | N/A | N/A | N/A | Data collection (citizens collected and submitted mosquitoes) | open call (nationwide invitation to public) | Surveillance - species identification (monitor indoor mosquito diversity) | General mosquito monitoring (vector species mentioned but not disease-specific) | Web portal (Mückenatlas) | mosquito species (physical specimens morphologically and genetically identified) | Expert/laboratory validation of citizen-collected physical specimens; mosquitoes submitted to the ‘Mückenatlas’ project from inside private homes; samples were identified morphologically to species level by the involved institutes using standard keys, and ambiguous cases were genetically identified by CO1 barcoding; no formal citizen-versus-expert accuracy metric was reported | NMDS, PERMANOVA, PERMDISP, rarefaction, effective Shannon diversity, Chi-square tests | Bias acknowledged with simple statistical adjustment for unequal sampling effort; community composition, diversity, and urbanization-preference outputs; the study explicitly states that opportunistic citizen-science data were spatially biased, especially by population density and uneven sample sizes across urbanization groups, and it addressed this mainly by using rarefied species richness, effective Shannon diversity robust to unequal sample sizes, and weighted expected counts in chi-square tests to approximate sampling effort, rather than modelling reporting probability or detection bias directly | risk-factor identification (associations with urbanization levels) | participation bias, data bias (uneven spatial coverage, opportunistic sampling) | Sampling bias; Spatial bias; Validation limitation; Representativeness/generalizability issue | not applicable; privacy protection (dataset cannot be publicly released) | Mückenatlas uses an explicit consent/data-protection framework: participants submit with consent, personal data are handled confidentially, mosquito and location data are shared with research partners in anonymized form, contributors appear on the collector map only if they opt in, may use a pseudonym, and can withdraw consent later. | Not publicly available (privacy restrictions) |
| [51](https://doi.org/10.1038/s41598-020-80365-3) | Drivers of spatio-temporal variation in mosquito submissions to the citizen science project 'Mückenatlas' | Nadja Pernat | 2021 | Germany | N/A | Urban | Descriptive observational study (study describes dataset patterns and applies statistical modeling) | 21,768 submissions (total number of mosquito submissions 2012–2017) | N/A | N/A | N/A | Data collection (citizens collect and submit physical mosquito specimens) | Open call (national call for participation; any citizen can submit via website form) | Surveillance (purpose is to monitor mosquito presence and distribution, species occurrence, patterns) | General mosquito monitoring (covers multiple invasive and native mosquito species) | Web portal (Mückenatlas) | Mosquito species; text (participants submit physical specimens and information via form) | Expert/laboratory validation of citizen-collected physical specimens; ‘Mückenatlas’ participants caught mosquitoes and mailed physical samples with submission forms, and the project experts then identified specimens to species level using a standardized protocol, morphologically in routine cases and genetically in difficult cases such as damaged specimens or cryptic species; no formal citizen-versus-expert accuracy metric was reported | Hurdle models, VIF, descriptive statistics (paper applies binomial + truncated negative binomial models and exploratory analysis) | Explicit analytical investigation of participation/reporting bias; submission-count and submission-probability outputs; the study was specifically designed to identify biases induced by opportunistic citizen-science collection, and it modelled the spatial distribution and number of submissions with hurdle models using anthropogenic and environmental predictors such as human population, former East/West Germany, water presence, precipitation, and wind speed, concluding that most effects on submission patterns were mainly associated with participant recording behaviour | Trend analysis; spatial predictions (models identify spatial–temporal drivers and submission distributions) | Participation bias, data bias, generalizability (uneven spatial coverage, media-driven spikes, anthropogenic bias all described explicitly) | Sampling bias; Spatial bias; Recruitment/engagement challenge; Representativeness/generalizability issue | Consent obtained; privacy protected under GDPR (paper states anonymized data, GDPR compliance, voluntary submission) | Mückenatlas uses an explicit consent/data-protection framework: participants submit with consent, personal data are handled confidentially, mosquito and location data are shared with research partners in anonymized form, contributors appear on the collector map only if they opt in, may use a pseudonym, and can withdraw consent later. | <https://doi.org/10.4228/ZALF.DK.153> |
| [52](https://doi.org/10.3390/s22020695) | MOSQUITO EDGE: An Edge-Intelligent Real-Time Mosquito Threat Prediction Using an IoT-Enabled Hardware System | ShyamPolineni | 2022 | USA | Nationwide | Urban/rural | descriptive (model development + evaluation study) (study focuses on developing & evaluating an ML model) | N/A | N/A | N/A | N/A | Data analysis (citizen scientists only contributed larval data indirectly through GLOBE) | volunteers | Surveillance (presence/absence, larvae abundance from GLOBE) (because citizen data represented mosquito surveillance inputs) | General mosquito monitoring (model predicts mosquito presence globally) | Sensor, GPS; GLOBE Observer (Grove sensors + Raspberry Pi + smartphone GPS) (edge device uses sensors + GPS smartphone input) (from IoT device description) | larvae count, mosquito species (GLOBE larvae counts + NEON species-level presence/absence) | No explicit study-specific validation of citizen observations reported; citizen-science mosquito data from NASA’s GLOBE Observer were combined with professionally collected NEON trap data; GLOBE larval-abundance observations were converted into a binary presence/absence label using a threshold (>25 larvae = presence, <25 = absence), but the paper did not describe expert verification of the citizen observations, who validated them, or any identification-accuracy assessment for the citizen-science records themselves | Random Forest; regression; feature importance; confusion matrix (explicitly described statistical tools) | No explicit analytical correction for citizen participation/reporting effort; species-distribution / mosquito-threat prediction modelling; the study combined citizen-science GLOBE data with NEON observations and addressed class imbalance by using quality absence data in an amount equal to presence data, but it did not model reporting intensity, observation probability, detection bias, or spatial sampling effort in the citizen-science data | Hotspot maps, Risk models, Spatial predictions (model outputs global threat levels and spatial hotspots) | data bias (citizen science species bias), algorithm limitations (model accuracy), generalizability (global predictions limited), participation bias (GLOBE coverage uneven) | Data quality issue; Representativeness/generalizability issue; Technology/access barrier | privacy, data ownership (uses public citizen science datasets but no personal data; IRB not required) | GLOBE Observer uses account-based participation under NASA/GLOBE privacy rules; users register with an email address and country, public data are linked to a screen name rather than personal identifiers, email addresses are not publicly displayed, and photos are screened or blurred for faces/text before entering the database. | No direct dataset link (GLOBE API + NEON datasets cited) |
| [53](https://doi.org/10.1016/j.actatropica.2025.107810) | A 3-year entomological cluster randomised controlled trial to assess the efficacy of mass-trapping for Aedes albopictus control in France: The vectrap project | Paulina A. Pontifes | 2025 | France | N/A | Urban | Intervention; cluster randomized controlled trial (CRCT) (study explicitly describes an RCT evaluating a mass-trapping intervention) | N/A | N/A | N/A | N/A | Monitoring interventions; engagement in research (residents participated by hosting and maintaining traps in Year 3) (justification: Year 3 shifted to community-based trap maintenance) | Volunteers; community engagement (residents voluntarily allowed traps to be installed; field teams recruited households) | Vector control (purpose was to reduce Aedes albopictus populations via trapping) (intervention explicitly targets mosquito abundance reduction) | Aedes albopictus; is vector of these diseases; study focuses on its control; text references outbreaks) | Sensor; smart trap hardware (uses BG-Mosquitaire and BG-GAT traps—physical sensors, not apps/web platforms) | Mosquito species counts; trap captures (adult female Ae. albopictus) (justification: outcome measures = abundance from traps) | Not applicable / independent entomological monitoring rather than validation of citizen-submitted records; this study did not use a citizen-science platform where participants submitted mosquito photos, observations, or specimens for classification. Instead, mosquito outcomes were measured through standardized BG-Sentinel trap collections operated within the trial, and all collected mosquitoes were morphologically identified in the laboratory using taxonomic keys and the MosKey Tool | GLMM; GAMM; negative binomial models; likelihood ratio tests (statistical tools explicitly stated) | Not applicable for citizen-reporting bias; structured intervention trial with standardized entomological sampling and analytical control of sampling effort; mosquito abundance was measured by repeated BG-Sentinel trapping in both treated and control blocks, analysed with negative-binomial mixed models, and when pooling data across years the models included an offset for the number of trapping days, but the study did not address citizen participation/reporting effort because it did not rely on opportunistic citizen-submitted surveillance data | Risk-factor identification; trend analysis (analysis examined environmental predictors and intervention effects across years) (justification: post-treatment differences + environmental covariates) | Participation bias; implementation variability; generalizability (study reports variation in community participation, trap malfunction, inconsistent coverage) | Recruitment/engagement challenge; Data quality issue; Representativeness/generalizability issue | Consent obtained from residents for trap installation; privacy maintained (residents gave permission for trap placement—implied consent; no personal data collected) | Not applicable as a platform-level ethics field. This was a project-specific field intervention, not a standing app/web platform with its own standard user-agreement, privacy, or data-ownership framework described in the paper. Resident consent was obtained for trap installation on private properties. | <https://figshare.com/s/eaa8c66e1dbf7fe9b379> |
| [54](https://doi.org/10.1007/s00436-023-08106-9) | A citizen science report—Tiger mosquitoes (Aedes albopictus) in allotment gardens in Graz, Styria, Austria | Julia Reichl | 2024 | Austria | N/A | Urban | descriptive observational study (study describes citizen-reported detection & monitoring) | N/A | N/A | N/A | N/A | Data collection (Level of Citizen Involvement, citizens collected adult mosquitoes and operated ovitraps) | volunteers (involvement began when a garden owner and residents contacted experts; no formal recruitment) | Surveillance (species identification, presence/absence, trap monitoring) (citizens trapped adults and monitored ovitraps) | Aedes albopictus (dengue, Zika, chikungunya) (Aedes albopictus is highlighted as a vector for these) | smart phone, Sensor (ovitrap monitoring + citizen reporting; tools are physical traps and citizen reporting) | larve count, mosquito species (ovitrap egg counts, adult mosquito specimens identified morphologically/molecularly) | Expert/laboratory validation with morphological identification and molecular DNA barcoding; citizen scientists collected adult mosquitoes and operated ovitraps, and eggs plus adults were sent to the laboratory for stereo-microscope examination, morphological identification, and COI-based DNA barcoding; most positive ovitrap findings were confirmed by morphology and molecular analysis, while some egg samples were identified morphologically only because barcoding quality was poor; adult haplotype analysis was also performed on a subset of specimens | descriptive statistics only (Statistical analysis — justified: counts, presence/absence, no inferential statistics) | Not applicable / no explicit analytical correction for participation effort; targeted presence-and-breeding detection study rather than bias-sensitive quantitative surveillance analysis; the study used ovitrap monitoring at three locations and citizen-collected adults to document the presence and breeding of Aedes albopictus in Graz, but it did not model reporting intensity, observation probability, or sampling-effort bias, and its main conclusions were based on confirmed detection rather than abundance, hotspot, or temporal-trend estimates | Trend analysis | participation bias, data bias, generalizability (single-city observations, limited locations, citizen-driven detection may underrepresent wider areas) | Validation limitation; Representativeness/generalizability issue; Data quality issue | Consent obtained from citizen scientists (article states no ethics approval required; participation was voluntary citizen reporting) | Not applicable as a platform-level ethics field. This was a project-specific monitoring approach, not a standing app/web platform with its own standard user-agreement, privacy, or data-ownership framework described in the paper. | Available upon reasonable request |
| [55](https://doi.org/10.1089/tmj.2023.0200) | A Smartphone App for Real-Time Assessment of Malaria Prophylaxis Adverse Events | Natalia Rodriguez-Valero | 2024 | Spain | N/A | Urban | Descriptive observational study (single-center observational evaluation) | 604 travelers | Median age 34 years | 55.2% female | N/A | Data collection (participants used app for real-time symptom reporting) | Volunteers (travelers invited during pre-travel consultation) | Surveillance; symptom reporting for malaria prophylaxis (real-time monitoring purpose) | Malaria (focus on malaria prophylaxis adverse events) | Mobile app, smartphone (Trip Doctor app used by participants) | Text; symptom reports (daily/weekly symptom entries) | Not applicable / not relevant to mosquito-surveillance record validation; this study used the Trip Doctor smartphone app for real-time reporting of traveler symptoms and adherence to malaria prophylaxis, not for mosquito observation, specimen, image, or vector classification. The app monitored health status, captured symptom questionnaires, and allowed physicians to monitor participants remotely, but the paper did not describe any mosquito-data validation workflow | Descriptive statistics, Wilcoxon test, chi-square tests | Not applicable; health-symptom reporting study rather than mosquito citizen-science surveillance-output analysis. The paper compared travelers prescribed versus not prescribed malaria prophylaxis, analyzed symptom questionnaires, and found no significant relationship between number of malarial pills taken and reported symptoms, but it did not address mosquito reporting intensity, sampling effort, detection bias, or participation bias in ecological/vector-surveillance outputs | Trend analysis (comparison of symptom occurrence across groups) | Participation bias, missing data, limited generalizability | Data quality issue; Representativeness/generalizability issue | Consent obtained; digital informed consent and privacy protections (ethics section) | No clear public platform-level ethics framework was reported; ethics were mainly project-level, including digital informed consent, privacy and legal disclaimers, ethics committee approval, and secure cloud/web-based data storage and monitoring. | Not available |
| [56](https://doi.org/10.2987/18-6789.1) | USING CITIZEN SCIENCE TO ENHANCE SURVEILLANCE OF AEDES AEGYPTI IN ARIZONA, 2015-17 | KARA D. TARTER | 2019 | USA | Arizona | Urban/rural | descriptive surveillance study (multi-year citizen-science monitoring) | 120 groups (schools and youth organizations) | N/A | N/A | N/A | Data collection (citizens deployed ovitraps and returned egg sheets) | open call; and automatic eN/Aollment (kits mailed statewide, online registration later) | surveillance (species identification and presence/absence) (Aedes egg detection) | Aedes aegypti (focus vector) | ovitrap kits and printed instructions (physical trapping tools, no digital tech) | mosquito species (eggs on germination paper used to identify Aedes aegypti presence) | Expert validation of citizen-collected ovitrap samples, with species confirmation by rearing; participating schools and youth groups deployed ovitraps and mailed egg sheets to collaborating entomologists, who verified whether *Aedes* eggs were present and reared a selection of eggs to adults for species identification; all successfully hatched eggs were identified as *Aedes aegypti*; no formal validation-accuracy metric was reported, but the study noted many participant false positives when visually reporting eggs, while no false negatives were reported | chi square analysis (statistical test used) | No explicit analytical correction for participation/reporting effort; detection-oriented surveillance outputs that are less sensitive than abundance estimates; the study mainly used verified ovitrap positives to identify *Aedes aegypti* occurrence and a few new activity areas, compared participation and positive catches across years, and interpreted results in relation to project timing, trap procedures, and routine surveillance, but it did not model reporting intensity, observation probability, or sampling-effort bias statistically | risk-factor identification (the project identified new areas of Ae. aegypti activity that were missed by routine surveillance) | articipation bias (uneven participation across years), data bias (incorrect egg identification by participants), generalizability (limited accuracy due to protocol variability) | Recruitment/engagement challenge; Data quality issue; Validation limitation; Spatial bias | no formal ethics review required (program evaluation not human-subject research), | Not applicable as a platform-level ethics field. This was a project-specific surveillance/education program, not a standing app/web platform with its own standard user-agreement, privacy, or data-ownership framework described in the paper. The paper notes that formal human subjects review was not required. | <https://www.azdhs.gov/preparedness/epidemiology-disease-control/mosquito-borne/index.php> |
| [57](https://doi.org/10.1186/s12942-023-00350-7) | Global mosquito observations dashboard (GMOD): creating a user-friendly web interface fueled by citizen science to monitor invasive and vector mosquitoes | Johnny A. Uelmen Jr. | 2023 | Global (platform covers global data from 127+ countries) | N/A | Urban/rural ( because GMOD aggregates submissions from many countries and environments) | Descriptive / methodological ( system development, data integration, and platform characteristics) | N/A | N/A | N/A | N/A | Data collection (citizen scientists submit mosquito observations, habitats, bites) (paper states GMOD is fueled by citizen observations) | Open call (participation via public apps like GLOBE Observer, Mosquito Alert, iNaturalist) (freely available apps; anyone can participate) | Surveillance (species identification, habitat reporting), education (platform used as a public learning tool) | General mosquito monitoring (focus on multiple vector and invasive species including Aedes, Culex, Anopheles) | Mobile apps, Web portal, GPS, smart phone (GMOD built from data generated via smartphone apps with GPS-enabled photos) | Images (photo), text, mosquito species, breeding sites (submissions include photos, habitat descriptions, larvae/pupae counts) | Platform-integrated, mixed validation framework; no new GMOD-specific validation workflow was performed by this study itself. Instead, GMOD aggregates data from platforms that use different validation approaches: Mosquito Alert adult-photo submissions are validated by a team of entomologists, iNaturalist observations rely on community/crowdsourced identification with “research grade” agreement and AI-assisted suggestions, and GLOBE Mosquito Habitat Mapper guides users through a visual key for larval genus identification; no overall cross-platform validation-accuracy metric was reported for GMOD in this paper | None | Participation/reporting bias explicitly discussed at the platform/global level, but not formally corrected analytically in this study; dashboard/integration platform rather than a stand-alone ecological-output modelling study. The paper states that reporting gaps exist by world region, socioeconomic status, and urban-versus-rural context, and that citizen-science point data are more strongly correlated with human population density and smartphone access than with mosquito abundance, but GMOD does not itself apply a formal adjustment for reporting intensity, observation probability, or sampling effort in the integrated dashboard outputs | Trend analysis, Spatial predictions, Risk-factor identification (GMOD enables visualization of trends and supports modeling of distributions and risks) | Participation bias, generalizability, data bias (paper states geographic and socioeconomic gaps; data correlated with human density, not mosquito abundance) | Sampling bias; Spatial bias; Representativeness/generalizability issue | No personal data; no consent needed; privacy maintained (no protected human data were used and participation involves open citizen science data) | GMOD is an open-access aggregation platform; platform-level ethics are largely inherited from the source platforms rather than newly defined by GMOD itself. The paper emphasizes open data access, data sharing, and reuse, and states that protected human or animal data were not used. | https://www.mosquitodashboard.org (free public data available under the “Data” tab) |
| [58](https://doi.org/10.1016/j.scitotenv.2024.174847) | Involving citizen scientists in monitoring arthropod vectors of human and zoonotic diseases: The case of Mosquito Alert in Italy | C.Virgillito | 2024 | Italy | N/A | Urban/rural | Descriptive and evaluation study (evaluates recruitment, engagement, and performance patterns; analyzes communication impact) | 18,291 registered participants; ~6,180 active reporters | N/A | N/A | N/A | Data collection (citizens submitted mosquito photos, bite reports, and breeding site records) | Open call recruitment via media and public outreach (recruitment mainly through press releases, newspaper articles, TV/radio interviews, local events) | Surveillance (species identification, presence/absence, breeding sites) (reports include mosquito photos, bites, breeding sites; purpose is mosquito monitoring) | General mosquito monitoring (Aedes albopictus, Aedes koreicus, Aedes japonicus, Culex pipiens) | Mobile app, GPS, smartphone (Mosquito Alert is a smartphone app using georeferenced reporting and optional GPS location sampling) | Image (photo), text, breeding sites, mosquito species (citizens submit photos of adult mosquitoes, text-based bite reports, and breeding site records) | Expert validation of citizen-submitted adult mosquito photos through the Mosquito Alert Entolab system, with quantified participant-versus-expert agreement; photographed adult mosquito records were identified by Entolab experts whenever possible, and citizen classifications (“invasive *Aedes*” vs. “*Culex* spp.”) were compared with expert labels. Overall, 86% of citizen identifications agreed with experts; agreement was 94% for records labelled by participants as invasive *Aedes* and 74% for those labelled as *Culex* spp., and participant identification accuracy improved with app use from about 61% initially to 75% after ~300 days | GLMM, GAM, GAMM, descriptive statistics | Explicit analytical modelling of participation/reporting effort at platform level; the study used optional anonymous masked background locations from participants to estimate inferred sampling effort, modelled the probability of sending at least one record as a function of participation time, and then calculated spatial sampling effort for each grid cell as the summed reporting probabilities of active participants over the previous two weeks. The paper also notes that this sampling-effort information can be used to reduce sampling bias in estimating human–mosquito interactions | Trend analysis and risk-factor identification (models identify temporal trends, engagement decline, sampling effort patterns) | Participation bias, data bias, generalizability (uneven engagement, sampling bias, regional differences, short-term engagement issues) | Recruitment/engagement challenge; Sampling bias; Spatial bias; Validation limitation | Consent obtained; privacy protection; masked GPS locations (app requires consent, anonymizes and masks geolocation for privacy) | Mosquito Alert requires acceptance of its user agreement and privacy policy, supports anonymous participation, asks users not to include personal information, links records to protected/random participant identifiers rather than exact public identities, and shares submitted images/data under project and open-data licensing terms. | <https://doi.org/10.5281/zenodo.10649985> |
| [59](https://doi.org/10.1007/s44217-024-00293-6) | Incorporating citizen science engagement in a vector surveillance undergraduate internship | Helen Urpi Wagner‑Coello | 2024 | USA | Florida | Urban | Evaluation; educational intervention (program outcome assessment) | 312 | 18–24 (79.5%) | Majority female (60%) | Primarily Hispanic/Latino (80.2%) | Data collection; engagement in research activities (egg collection + training) | Open call; via email, courses, and internship announcements | Surveillance; education (egg collection, mosquito biology training) | General mosquito monitoring (contextual MDC mosquito-borne disease risk) | Smartphone, GPS-enabled reporting, ovitraps (ovicups used for egg collection) | Image (egg photos), mosquito egg counts, text survey responses | Expert analysis of citizen-collected ovitrap materials implied, but not described as a formal validation workflow; FLAGG interns collected mosquito eggs using ovicups in residential settings and sent photographs and egg papers back to the surveillance team experts for analysis, while also receiving training in egg counting, mosquito life stages, breeding habitats, and surveillance methods. However, this paper did not detail who validated each submission operationally, how species identification was confirmed, or any identification-accuracy metric | ANOVA, Brown-Forsythe, Welch, Tukey HSD, Games-Howell | Not applicable (education/internship outcomes study rather than citizen-surveillance-output analysis); the paper evaluated whether FLAGG participation improved mosquito-abatement knowledge, behavior competence, academic confidence, career-related confidence, and engagement, using surveys and group comparisons, rather than modelling mosquito abundance, hotspot patterns, temporal trends, or risk outputs from uneven citizen-reporting effort | None (no predictive or spatial models generated) | Participation bias and limited generalizability (university sample) | Representativeness/generalizability issue; Data quality issue | Consent obtained; IRB approval; privacy protections (IRB #IRB-22-0068) | Not applicable as a platform-level ethics field. This was a project-specific internship/surveillance program, not a standing app/web platform with its own standard user-agreement, privacy, or data-ownership framework. Ethics were handled at the project level through IRB approval and informed consent. | Not publicly available |
| [60](https://doi.org/10.1093/jme/tjx166) | The Citizen Science Project 'Mueckenatlas' Helps Monitor the Distribution and Spread of Invasive Mosquito Species in Germany | Doreen Walther and Helge Kampen | 2017 | Germany | N/A | Urban | descriptive passive surveillance (observational descriptive design using passive submissions | 7300 submissions (participant sample size: >7300 citizen submissions reported) | N/A | N/A | N/A | Data collection and monitoring interventions (citizens captured mosquitoes and submitted specimens, active data collection; monitoring reproduction sites triggered by submissions) | open call (recruitment through public media, press releases, newspapers, TV, flyers) | surveillance species identification distribution mapping (purpose: detect invasive species, update distribution maps, early warning system) | General mosquito monitoring including invasive Aedes (mosquito-borne disease covered: general monitoring + species relevant for dengue, chikungunya, West Nile via invasive Aedes) | Web portal (Mückenatlas) and manual specimen submission (citizens used the project website plus containers to physically send mosquitoes) | mosquito species specimens and text metadata (physical specimens plus collection metadata like location, time, environment) | Expert validation of citizen-submitted physical mosquito specimens, with morphological identification and genetic confirmation when needed; citizens mailed collected mosquitoes to the Mueckenatlas team, specimens were identified morphologically using taxonomic keys, and cryptic or damaged specimens were identified genetically by species-specific PCR or DNA barcoding. Invasive mosquitoes were usually also confirmed genetically, and the reported success rate of genetic identification was >95% | None | No explicit analytical correction for participation/reporting effort; passive surveillance used mainly for presence/distribution detection and as a trigger for targeted field follow-up rather than for abundance estimation. The paper notes that submission numbers may vary with mosquito abundance as well as media coverage, but it does not model reporting intensity, observation probability, or sampling effort statistically; instead, invasive-species submissions were followed by active field monitoring to verify local reproduction and wider distribution | spatial distribution mapping and detection of new populations (mapping invasive species distributions and new population detection) | participation bias misidentification risk uneven regional submissions (submissions depend on citizen motivation + accuracy) | Sampling bias; Recruitment/engagement challenge | data ownership privacy minimal risk (ethics: voluntary submissions; no personal data except optional name on map; no formal ethics due to passive, non-human-subjects data) | Mückenatlas uses an explicit consent/data-protection framework: participants submit with consent, personal data are handled confidentially, mosquito and location data are shared with research partners in anonymized form, contributors appear on the collector map only if they opt in, may use a pseudonym, and can withdraw consent later. | [www.mueckenatlas.de](http://www.mueckenatlas.de/) |
| [61](https://doi.org/10.52707/1081-1710-47.1.133) | First record of the mosquito Aedes (Downsiomyia) shehzadae (Diptera: Culicidae) in Australia: A unique discovery aided by citizen science | Cameron Webb | 2022 | Australia | Queensland | Urban/rural | Descriptive (the study documents a species record) | photographic contributor (one citizen uploaded the observation) | N/A | N/A | N/A | Data collection (citizen provided photographic evidence through iNaturalist) | Open call (iNaturalist allows anyone to upload observations) | Surveillance, species identification and presence documentation (the citizen observation enabled first national record) | General mosquito monitoring | Web portal (iNaturalist platform used for submission) | Image (photo) | Expert photo-based taxonomic assessment, but without specimen collection or molecular confirmation; a citizen scientist photographed an unusual adult mosquito and uploaded the image to iNaturalist, where it could not be readily identified by users and was then investigated by experienced mosquito researchers using taxonomic keys, existing records, and photographs of the Aedes shehzadae type specimen from the Natural History Museum, London. The authors concluded the photographed mosquito best matched Ae. shehzadae, but explicitly noted that, without additional specimens for morphological and genetic analysis, complete confirmation was not possible | None (no statistical tests or modelling performed) | Not applicable / no explicit analytical correction for participation or reporting effort; first-record, presence-only discovery study rather than quantitative ecological-output analysis. The paper documents a single unusual mosquito observation uploaded through iNaturalist and uses it to report a possible first Australian record of *Ae. shehzadae*, while discussing citizen science as a useful complement to formal surveillance for detecting rare or exotic mosquitoes outside the scope of routine programs, but it does not model observation probability, sampling effort, reporting intensity, or spatial bias | None | Participation bias and identification uncertainty (identification based only on photograph) | Validation limitation | Implied consent through public upload; no personal data collected (no formal ethics review required) | iNaturalist uses account-based public participation; observations are publicly shared by default, location visibility can be restricted through geoprivacy settings, and users retain ownership of their content while licensing it under iNaturalist’s platform terms (default CC BY-NC unless changed). | <https://www.inaturalist.org/observations/76876488> |
