# Supplementary material for: The role of citizen science in mosquito-borne disease surveillance and control: A scoping review
Source: PLoS One. 2026 May 15;21(5):e0348697. doi: 10.1371/journal.pone.0348697 (PMC13178890; doi:10.1371/journal.pone.0348697)
Supplement: Attachment — Submitted filename: Response to Reviewers.docx [file pone.0348697.s003.docx]

Dear Editor and Reviewers,

Thank you for your time and careful review of our manuscript. We have thoroughly revised the manuscript in response to your valuable comments. We believe the revised version is substantially improved in clarity, organization, and presentation. Below, we provide a point-by-point response to each comment and indicate where revisions were made in the manuscript.

Sincerely,

**************************************************************************************

**Response to Editor**

**COMMENT**

After editorial assessment and external review, I am writing to let you know that the decision is major revision. The reviewers and I feel that the topic is relevant and the manuscript has clear potential, but several substantive issues need to be addressed before it can be considered further.

Please revise the manuscript carefully in light of the reviewers’ comments and provide a clear, point-by-point response explaining how each issue has been addressed.

**RESPONSE**

Thank you for your guidance and for the opportunity to revise our manuscript. We have carefully addressed reviewers’ comments and provided a detailed point-by-point response explaining how each issue was handled. All changes are highlighted in the revised manuscript.

**************************************************************************************

**Responses to Reviewer #1**

**Comments to the Author**
The paper provides a scoping review of studies incorporating citizen science elements for mosquito surveillance. The work offers a useful framework through which to understand global variation and biases in citizen science data within the field of mosquito-borne diseases (MBDs). The authors provide clear rationale for the work and the inclusion criteria are justified. The analysis is comprehensive and includes many parameters to identify differences and commonalities of studies. Statistical analysis is limited to percentages but is appropriate for the objectives. The authors find biases in spatial distribution of studies, with the majority of studies occurring in the western hemisphere and the Global North. The work adds to existing literature on the topic of citizen science for vector surveillance through the inclusion here of ethical considerations and participation models, as well as useful discussion of spatial and methodological biases. The paper is written well with clear objectives, methodology, findings and conclusions. All supporting data are available within the paper and supplementary information.

**RESPONSE**

We sincerely thank Reviewer #1 for their time and valuable feedback. Their insightful comments have significantly contributed to improving the quality of our manuscript. In this revised version, we have provided specific responses to each comment and highlighted all corresponding changes in the text.

******

**COMMENT**

Minor comments:

Line 172-181: Listing the numbers of countries that contributed studies from each continent may cause confusion as this is not reflected in the map in Figure 2, where far more countries are included due to multi-country studies - this is made clear in the supporting information but is not explicit in the manuscript. It would be helpful to mention the total number of countries from which citizen science studies were included, either in the same paragraph or within the figure legend for Figure 2.

**RESPONSE**

Thank you for this helpful suggestion. We agree that this point required clarification and have revised both the *Results* section 3.2 and the *Figure 2* caption accordingly:

Added text to *Results* section 3.2:

“Overall, the included studies covered 24 unique countries.”

Added text to *Figure 2* caption:

“Panel A shows all countries represented across the included studies (n=24 unique countries), including those captured through multi-country study designs.”

******

**COMMENT**

Line 194-195: It might be useful to clarify how species were determined to be invasive – was this based on studies that specifically mentioned invasive species monitoring? If not, was the definition of ‘invasive’ accounted for across studies that may have included the native range of ‘invasive’ species e.g. Aedes aegypti?

**RESPONSE**

Thank you for this important recommendation. We revised *Results* section 3.3 to clarify that this classification was based on how the original studies described the target species within their specific geographic and surveillance contexts, and revised the wording accordingly:

*Results* section 3.3:
“Among the included studies, mosquito monitoring was the most common focus (n=40, 65.6%), primarily concentrated on mapping the distribution, abundance, and spread of mosquito taxa, including species described by the original studies as invasive or nuisance in specific geographic and surveillance contexts. *Aedes albopictus* was the most commonly reported species (n=16, 26.2%)^2,7,10,15,22,24,26–28,35–40,45^, followed by *Aedes japonicus* (n=6, 9.8%)^2,23,25,26,38,39^, *Aedes koreicus* (n=5, 8.2%)^2,10,32,38,39^, and *Aedes aegypti* (n=3, 4.9%)^2,26,47^. Additional species included *Aedes notoscriptus* (n=1, 1.6%)^66^ and *Culex* species, most commonly *Culex pipiens* (n=4, 6.6%)^2,10,35,41^ and *Culex quinquefasciatus* (n=1, 1.6%)^66^.

A smaller subset of studies focused on *Anopheles* species, including *Anopheles plumbeus* (n=2, 3.3%)^30,31^, members of the *Anopheles gambiae* complex (*A. gambiae* s.s., *A. arabiensis*, *A. funestus;* n=1, 1.6%)^60^, and mixed *Anopheles* larvae observations through community surveillance platforms (n=3, 4.9%)^11,74,75^.”

**

**COMMENT**

Line 211: Maybe the number of studies from the Americas and Oceania that included surveillance for dengue/ Zika/ Chikungunya could also be provided, for consistency.

**RESPONSE**
Thank you for this important observation. We revised *Results* section 3.3 to provide the corresponding counts for studies from the Americas and Oceania that focused on surveillance targeting dengue, Zika, or chikungunya.

*Results* section 3.3:

“Several studies from the Americas (n=5 of 13, 38.5%) and Oceania (n=2 of 7, 28.6%) also focused on dengue, Zika, or chikungunya surveillance.”

******

**COMMENT**

Line 240: It would be interesting to know what other traps were used and the breakdown of trap types by number of studies.

**RESPONSE**

Thank you for this helpful suggestion. We revised *Results* section 3.5.1 to provide the breakdown of trap types used across the included studies:

*Results* section 3.5.1:

“Trap-based data collection was also reported in 11 studies (n=11, 18.0%), most commonly using ovitraps (n=5 of 11, 45.5%). Other trap types included BG-Sentinel traps (n=3, 27.3%), BG Gravid *Aedes* Traps (BG-GAT) (n=3, 27.3%), CO₂-baited traps (n=2, 18.2%), and BG-Mosquitaire and CDC light traps (n=1 each, 9.1%). Some studies used more than one trap type, so the total number of trap occurrences exceeds the number of studies.”

******

**COMMENT**

Line 271-272: Maybe the last three approaches can be reordered to reflect decreasing frequency (public websites, social media campaigns, institutional newsletters).

**RESPONSE**

Thank you. We revised the order of the last three recruitment approaches in *Results* section 3.5.5 to reflect decreasing frequency:

*Results* section 3.5.5:

“…public websites or email invitations (n=5, 8.2%)^26,27,30,35,44^, social media campaigns (n=4, 6.6%)^10,45,61,63^, and institutional newsletters or academic channels (n=3, 4.9%)^44,61,63^.”

******

**COMMENT**

General comment: The discussion lists several biases of citizen science data. While not necessary, it could be interesting to give an overview (in the results) of biases that the studies themselves have identified in their work.

**RESPONSE**

We thank the reviewer for this helpful suggestion. We agree and added a new *Results* subsection, Section 3.9 *(“Reported biases and methodological limitations”*), to summarize the main biases and limitations explicitly reported by the included studies. We also revised *Methods* Sections 2.4 and 2.5, and the *Abstract* accordingly:

*Results* section 3.9 (added):

“3.9 Reported biases and methodological limitations

Across the included studies, several explicitly reported biases or methodological limitations affecting their own citizen science data or implementation. Data quality issues were the most common (n=35, 57.4%), followed by validation limitations (n=32, 52.5%), recruitment/engagement challenges (n=32, 52.5%), and participation and sampling biases reported by the studies themselves (n=27, 44.3%), which included uneven observation or reporting effort. Representativeness limitations, indicating that the contributed data may not adequately reflect the full target population or study context, were noted in 25 studies (n=25, 41.0%). Technology-related barriers (n=16, 26.2%) and spatial bias (n=15, 24.6%) were reported less often.”

*Methods* section 2.4 (revised):

“…v) reported biases, methodological limitations, and ethical considerations (e.g. participation and sampling biases, data quality issues, validation limitations, recruitment/engagement challenges, representativeness limitations, technology-related barriers, spatial bias, ethical procedures, and data availability).”

*Methods* section 2.5 (revised):

“…reported biases and methodological limitations, and ethical considerations.”

*Abstract* section (revised):

“…reported biases and methodological limitations, and ethical and governance practices.”

******

**############################################################################**

**Responses to Reviewer #2**

**Comments to the Author**

This manuscript presents a scoping review examining the role of citizen science in mosquito-borne disease surveillance and control. The authors analyze 61 studies published between 2000 and 2025 that use citizen participation to collect mosquito-related data or support vector surveillance initiatives. The review summarizes study characteristics, technologies used, participation strategies, analytical outputs, and governance practices. The topic is timely and highly relevant. Citizen science initiatives have become an increasingly important complement to traditional mosquito surveillance programs, particularly with the widespread availability of mobile technologies and digital reporting platforms. The manuscript provides a useful overview of existing initiatives and highlights the rapid growth of citizen-science projects in this field. However, several aspects that determine the scientific robustness and operational value of citizen-science surveillance systems are insufficiently addressed in the analysis and would benefit from further development.

**RESPONSE**

We sincerely thank Reviewer #2 for their thoughtful summary and positive evaluation of our work. We are grateful for the constructive feedback which has contributed to strengthening the overall quality and rigor of our study. In the revised manuscript, we have carefully addressed the reviewer’s suggestions and incorporated additional clarifications where appropriate. Major changes have been highlighted for ease of review.

******

**COMMENT**

A first aspect concerns the discussion of ethical and governance issues. In the current manuscript, governance considerations are mainly addressed in terms of whether ethical procedures are reported by the studies included in the review. While this is important, the discussion could be expanded to consider the role of ethical transparency as a mechanism to foster participation and trust among citizens. Citizen-science initiatives depend fundamentally on voluntary engagement from the public, and participation levels are strongly influenced by the degree of trust participants place in the project. Clear communication about how data are collected, how they will be used, and how personal information is protected is, therefore, not only an ethical requirement but also a practical mechanism to encourage participation. In many mosquito citizen-science platforms, personal data collection is deliberately minimized or avoided, and the explicit communication of this principle can serve as an important incentive for participation. Addressing how different projects communicate these governance and privacy practices to participants would therefore provide a more nuanced perspective on the role of ethics and governance in citizen-science initiatives. Linked to that, as the manuscript highlights, many studies are related to the same citizen science project or use the same platform, and authors should make sure that in all these cases, some basic information related to ethical or data collection issues should not be mentioned, considering that it has been previously described or explained in previous papers. Saying that to make note of the authors to check for consistency for platforms or projects beyond what is explained or described in a single paper. For example, checking Supplementary file 2 “Extraction table” it is possible to see that kind of inconsistencies between study 1 and 2, both use Mosquito Alert tools, in the 1st ethics about the obtention of pictures and reports is stated (e.g. user agreement, anonymous participation, etc.), while in the 2nd, even if also use Mosquito Alert for image classification, the obtention of the images appears as “Not stated”. If they used the Mosquito Alert app to collect images, it is supposed that reports have been collected with user agreement and anonymous participation again. I will recommend the authors in that table to create a clear field identifying the project or tool used to unify the studies and check that information is consistent across all studies for the same platform and tool.

**RESPONSE**

We thank the reviewer for this thoughtful and important comment. We agree that the previous version focused mainly on whether ethical procedures were explicitly reported by individual studies and did not sufficiently consider ethical transparency as a mechanism for fostering trust and participation. We also agree that recurring platforms may embed governance and privacy features that are not restated in every publication. To address this, we revised the *Discussion*, expanded *Results* section 3.10, and updated S2 File by adding a platform-level ethics column and improving consistency across studies using the same platform. The following revisions were made:

*Results* section 3.10 (added):

“Notably, several recurring digital platforms operated under established governance frameworks that commonly addressed consent, privacy, anonymization, visibility, and data sharing. As these provisions were often embedded within the platform itself, they were not always restated explicitly in every individual study using the same tools. For example, Mosquito Alert incorporated user-agreement and privacy-policy acceptance, anonymous participation, random user identifiers, geolocation protection, and open-data or licensing terms. GLOBE Observer used account-based participation with public screen names (rather than public personal identifiers) and screened submitted photographs for faces or identifying text. Mückenatlas used explicit consent procedures, confidential handling of personal data, anonymized sharing of mosquito and location information, and opt-in public visibility. Similarly, iNaturalist allowed users to retain ownership of submitted content while selecting licenses and controlling geoprivacy settings for observations.”

*Discussion* section (added):

“Beyond reporting practices, ethical transparency plays a central role in citizen science systems by shaping participant trust, engagement, and sustained contribution. Because these initiatives rely on voluntary public participation, clear communication about what data are collected, how they are used, how privacy is protected, and whether submissions are anonymized or shared openly is not only an ethical requirement but also a practical mechanism for encouraging participation. Many mosquito-focused citizen science platforms deliberately minimize personal data collection, use anonymized or masked identifiers, or allow users to control visibility and sharing settings. When clearly communicated, these protections may therefore strengthen confidence in participation and support sustained data contribution. In this context, transparent and standardized reporting of governance practices would provide a better understanding of how citizen science systems foster trust while managing privacy, data use, and accountability.”

******

**COMMENT**

A second important issue relates to participation biases in citizen-science datasets. A well-known limitation of citizen-science initiatives is that participation intensity is strongly dependent on population density. Areas with larger populations tend to generate more observations simply because more potential contributors are present, whereas rural or sparsely populated areas often remain underrepresented. This urban participation bias has important implications for mosquito surveillance systems, particularly when citizen-generated data are used for spatial analyses such as hotspot detection, abundance estimation, or risk mapping. The manuscript notes that a large proportion of studies were conducted in urban environments, and in the discussion highlights the bias risk, but the analysis would benefit from exploring more explicitly how different projects address this structural bias. Some initiatives attempt to mitigate these imbalances through targeted recruitment strategies, for example, by organizing communication campaigns or partnerships aimed at engaging communities in rural or less populated regions where participation is otherwise more difficult to achieve. Examining whether and how such strategies are implemented across the reviewed studies would provide valuable insight into how citizen-science projects attempt to balance participation across different geographical contexts.

**RESPONSE**

Thank you for this helpful suggestion. We agree and added a new paragraph to *Results* section 3.5.5 to summarize how the included studies addressed structural participation imbalance operationally, and we revised the *Discussion* accordingly.

*Results* section 3.5.5 (added):

“Recruitment strategies also differed in how directly studies addressed structural participation imbalance across geographic contexts. In 33 studies (n=33, 54.1%), recruitment, engagement, or outreach approaches clearly or partly aimed to broaden participation beyond highly populated settings. Of these, seven studies (n=7, 11.5%) implemented more explicit targeted strategies for rural, remote, or otherwise underserved areas^32,42,47–50,57^. Within these seven studies, common approaches included partnerships with schools or school-based networks (n=6, 9.8%), engagement with community leaders, local authorities, or community health workers (n=6, 9.8%), door-to-door or household visits (n=3, 4.9%), village-based recruitment structures (n=2, 3.3%), and low-access tailored measures such as shared smartphones, offline data collection, or local-language outreach (n=1, 1.6%) (non-mutually exclusive). The other 26 studies (n=26, 42.6%) addressed this issue more indirectly through locally adapted outreach, media or social-media campaigns, expanded recruitment networks, or discussion of ways to strengthen participation in low-contribution regions, without clearly framing these efforts as explicit corrections for urban or population-density bias. In the remaining 28 studies (n=28, 45.9%), no clear recruitment-side strategy to reduce structural participation imbalance was reported. These patterns indicate that recruitment strategy was not merely an operational detail, but a component of system design that shaped who participated, where data were generated, and how vulnerable the resulting outputs were to structural participation imbalance.”

*Discussion* section (added):

“Consistent with this pattern, only a small subset of studies implemented clearly targeted recruitment strategies for rural, remote, or otherwise underserved settings. Most addressed this issue only indirectly through broader outreach or did not clearly report a recruitment-side strategy to mitigate structural participation imbalance.”

******

**COMMENT**

Closely related to this issue is the analytical treatment of participation biases in the studies reviewed. Citizen-science datasets inevitably reflect both ecological patterns and patterns of reporting activity, and this distinction is particularly important when data are used to generate quantitative outputs. Analyses based on citizen observations should ideally account for variations in participation effort, as differences in reporting intensity can strongly influence estimates of mosquito abundance, the identification of spatial hotspots, or the interpretation of temporal trends. Without appropriate corrections, such outputs may reflect patterns of participation rather than true ecological dynamics. The manuscript would therefore benefit from a more systematic examination of how the reviewed studies address sampling effort in their analyses. Some analytical approaches explicitly incorporate participation intensity or observation probability, whereas others use modelling frameworks designed to account for detection biases. At the same time, it is important to recognize that not all types of outputs are equally sensitive to participation bias. For example, detecting the presence of a mosquito species in a region is relatively independent of sampling effort, as the species is either detected or not detected. In contrast, estimates of abundance, spatial clustering, or temporal trends are much more sensitive to variations in participation effort. Distinguishing between these different types of outputs and discussing how they are affected by participation biases would significantly strengthen the analytical depth of the review.

**RESPONSE**

Thank you for highlighting this important point. We agree that the previous version did not examine the analytical handling of participation/reporting effort with sufficient depth. To address this, we added a new paragraph in *Results* section 3.8 to systematically summarize how the included studies handled effort bias, revised the *Discussion* section, updated *Methods* sections 2.4 and 2.5, and revised the *Abstract* accordingly. We also updated S2 File by adding a new extraction column, “Effort-bias handling in analysis,”. The following revisions were made:

*Results* section 3.8 (revised title and added text):
“3.8 Analytical approaches, effort-bias handling, and epidemiologic outputs
Handling of participation or reporting effort varied substantially across the included studies. In 23 studies (n=23, 37.7%), effort correction was not directly applicable because the analyses focused on intervention trials or structured entomological monitoring (n=9, 14.8%), co-design, implementation, or conceptual work (n=5, 8.2%), participation- or questionnaire-based non-ecological analyses (n=4, 6.6%), educational outcomes (n=2, 3.3%), image-classification studies (n=2, 3.3%), or non-vector reporting systems (n=1, 1.6%).

Explicit analytical adjustment or effort-aware modeling was identified in 11 studies (n=11, 18.0%). These included offsets for estimated sampling effort (n=2, 3.3%), weighted pseudo-absences or effort-weighted absences (n=2, 3.3%), effort-standardized abundance metrics (n=2, 3.3%), reporting-propensity models (n=2, 3.3%), and direct modeling of reporting behavior (n=1, 1.6%) (non-mutually exclusive).

In 10 studies (n=10, 16.4%), participation bias was examined, benchmarked, or partly accommodated without direct correction. These studies benchmarked citizen science records against external surveillance or reference datasets (n=4, 6.6%), filtered low-quality or anomalous records (n=1, 1.6%), or qualitative discussion of population-related reporting bias (n=9, 14.8%) (non-mutually exclusive). A further 10 studies (16.4%) focused on detection-oriented outputs (e.g., early detection, confirmed occurrence, range expansion) and did not apply formal effort correction. In seven studies (n=7, 11.5%), effort-related bias was partly mitigated through structured or standardized citizen-assisted sampling designs without separate analytical correction.

The impact of uneven reporting varied by outcome. Outputs based on species presence, first detection, or confirmed occurrence were generally less sensitive to uneven reporting intensity. In contrast, abundance estimates, hotspot mapping, spatial clustering, temporal trend analyses, and some risk-modeling outputs were more vulnerable to participation bias. Accordingly, the implications of effort bias varied across studies depending on the type of surveillance output generated.”

*Discussion* section (added):

“Only a minority of studies used explicit effort-aware modeling. Most either addressed participation bias qualitatively or focused on outputs less sensitive to uneven reporting, such as early detection or confirmed presence. This distinction is important because abundance estimates, hotspot mapping, spatial clustering, and temporal trend analyses are more vulnerable to participation bias than detection-oriented surveillance outputs.”

*Methods* section 2.4 (revised):

“…iii) MBDs focus, validation mechanisms, and analytical approaches (disease(s) studied, data types collected, validation procedures, effort-bias handling, analytical or modeling methods, and epidemiologic outputs) ...”

*Methods* section 2.5 (revised):

“The selected studies were categorized based on study characteristics, MBDs focus, citizen science approach, platforms and technologies used, validation mechanisms, effort-bias handling, analytical approaches, reported biases and methodological limitations, and ethical considerations.”

*Abstract* section (revised):

“Data were extracted and synthesized on study characteristics, participation objectives, recruitment strategies, citizen-generated data and technologies, validation mechanisms, effort-bias handling, analytical approaches, public health outputs, reported biases and methodological limitations, and ethical and governance practices.”

******

**COMMENT**

Another important dimension that appears to be largely absent from the current analysis concerns the validation of citizen-generated observations. The scientific reliability of citizen-science mosquito surveillance depends critically on how submitted observations are verified and classified. Different projects employ a range of validation mechanisms, including expert entomologist review, distributed networks of specialists, community validation processes, or automated classification tools based on artificial intelligence. These procedures can vary considerably in terms of accuracy, speed, and scalability, and they directly influence the reliability of the datasets generated by citizen-science platforms. The review would therefore benefit from examining how the different initiatives included in the study handle the classification and validation of mosquito specimens or images, who performs these validations, and what is known about the accuracy of the identification processes. Understanding these mechanisms is essential for assessing the scientific value of the outputs generated by the platforms.

**RESPONSE**
Thank you for this constructive comment. We agree that validation mechanisms are central to interpreting the scientific reliability of citizen-generated mosquito data. To address this point, we added a new *Results* subsection, Section 3.7 (“Validation mechanisms”), revised the *Abstract*, *Methods* Sections 2.4 and 2.5, and incorporated a concise interpretive statement in the *Discussion*. We also updated S2 File by adding a new extraction column, “Validation mechanism,” to capture how observations were verified, and whether any validation-performance information was reported. The following revisions were made:

*Results* section 3.7 (added):

“3.7 Validation mechanisms

Validation of citizen-generated observations was addressed in 48 studies (n=48, 78.7%). In the remaining 13 studies (n=13, 21.3%), validation was not applicable because the primary outcomes were derived from independent entomological monitoring or intervention assessments (n=9, 14.8%), conceptual or educational program design (n=3, 4.9%), or non-vector reporting systems (n=1, 1.6%), rather than from citizen-submitted records requiring taxonomic verification.

Expert-led validation was the dominant approach (n=33, 54.1%). These workflows included app- or photo-based expert review (n=16, 26.2%), laboratory processing of mailed specimens or ovitrap materials (n=15, 24.6%), and expert validation followed by field confirmation (n=2, 3.3%). Morphological examination, rearing, or molecular confirmation supported expert-led workflows in 11 studies (n=11, 18.0%).

Hybrid or mixed validation approaches were reported in six studies (n=6, 9.8%), typically combining automated classification, participant-assisted identification, or cross-platform workflows with subsequent expert review. Fully automated classification was uncommon (n=3, 4.9%) and largely limited to acoustic detection systems. Other validation approaches appeared only once each (n=1, 1.6%), including community-based classification, participant self-identification supported by voucher photographs, and independent entomological verification of community-ranked mosquito-density outputs. In three studies (n=3, 4.9%), validation procedures were not clearly described.

Validation approaches varied systematically by platform type. App- and image-based reporting systems most often relied on expert review or hybrid workflows, whereas specimen- and trap-based systems commonly used laboratory or field confirmation, reflecting differences in data type and validation demands created by platform design.

Quantitative validation performance was reported inconsistently. Five studies (n=5, 8.2%) reported expert–participant agreement or image-identification accuracy ranging from approximately 86% to 94%. One study (n=1, 1.6%) reported species-specific expert-confirmed accuracies of 87.6% for *Aedes albopictus* and 81.3% for *Aedes aegypti*, another (n=1, 1.6%) reported early-warning specificity of approximately 97%, and one (n=1, 1.6%) reported a ROC AUC of about 0.96 with approximately 98% accuracy for a large subset of images. Performance was more variable in one larval self-identification study (n=1, 1.6%), with accuracy ranging from 34% to 100% across countries, and an acoustic-classification study (n=1, 1.6%), where accuracy improved from approximately 35% to 65% after incorporating location metadata.”

*Discussion* section (revised):
“The review further suggests that the scientific value of citizen-generated mosquito data depends not only on the underlying validation workflow, but also on how participation model, effort-bias handling, and analytical objective are aligned. Low-burden public-reporting systems with strong expert validation were most often used for early detection, occurrence mapping, and spread tracking. In contrast, studies targeting quantitative outputs, such as hotspot mapping, temporal trend analysis, or risk modeling, more often required stronger validation, benchmarking, and effort-aware analytical approaches. Community-based initiatives with deeper local engagement more often oriented toward education, public engagement, and local vector-control activities. These roles were often overlapping rather than mutually exclusive.”

*Methods* section 2.4 (revised):

“…iii) MBDs focus, validation mechanisms, and analytical approaches (disease(s) studied, data types collected, validation procedures, effort-bias handling, analytical or modeling methods, and epidemiologic outputs) ...”

*Methods* section 2.5 (revised):

“The selected studies were categorized based on study characteristics, MBDs focus, citizen science approach, platforms and technologies used, validation mechanisms, effort-bias handling, analytical approaches, reported biases and methodological limitations, and ethical considerations.”

*Abstract* section (revised):

“Data were extracted and synthesized on study characteristics, participation objectives, recruitment strategies, citizen-generated data and technologies, validation mechanisms, effort-bias handling, analytical approaches, public health outputs, reported biases and methodological limitations, and ethical and governance practices.”

******

**COMMENT**

More broadly, and more importantly, from my point of view, the manuscript would benefit from adopting a more integrated analytical framework linking the different components of citizen-science systems. At present, the review discusses aspects such as data collection, participation strategies, analytical outputs, and governance largely as separate dimensions. However, the scientific value of a citizen-science platform ultimately depends on how these elements interact. A more synthetic perspective that connects the level of citizen participation, the validation procedures used, the analytical approaches applied, and the methods used to correct participation biases would provide a clearer picture of the strengths and limitations of different initiatives. Such an approach could also help identify distinct functional roles of citizen-science projects. Some platforms may primarily contribute to quantitative public-health surveillance by generating data that can be integrated into risk models, while others may focus more strongly on education and public engagement or on supporting early detection of invasive species. These different functions are not mutually exclusive, and no single model should necessarily be considered superior to others. However, distinguishing between them could help clarify the specific contributions that citizen-science initiatives can make to mosquito surveillance and public health. Developing this type of synthesis would significantly strengthen the manuscript by offering a more comprehensive overview of the diversity of citizen-science platforms and the different roles they play.

**RESPONSE**

Thank you for this insightful comment. We agree that the manuscript is strengthened by adopting a more integrated perspective on citizen science systems. In response, we substantially revised both the *Results* and *Discussion* sections to make the interrelationships among participation, validation, analytical outputs, bias handling, and governance more explicit, and to clarify the broad, overlapping functional roles of different citizen science initiatives. The following revisions were made:

*Results* section 3.4 (added):

“Study designs varied in how citizen science was used, ranging from descriptive surveillance to system evaluation and, less commonly, intervention and feasibility studies.”

*Results* section 3.5 (added):

“Citizen involvement varied both in intensity and in how directly it contributed to different surveillance functions, ranging from simple reporting of observations to more active roles in local monitoring, intervention, annotation, and basic analytic support.”

*Results* section 3.5.1 (added):

“In general, simpler citizen-contributed data types such as presence reports and photographs were commonly used to support occurrence mapping, species identification, and early detection. In contrast, trap-based, specimen-based, and acoustic data were more often used in structured monitoring or more specialized validation and analytic workflows.”

*Results* section 3.5.3 (added):

“Compared with observational reporting platforms, these studies positioned citizens as active co-implementers of surveillance and control, linking participation more directly to local vector management and public-health action.”

*Results* section 3.5.4 (revised):

“…in which citizen-contributed data (often photographic or app-based observations) were reviewed, labeled, or categorized to support species identification, model training, or quality control workflows. These annotation activities functioned as Human-in-the-Loop quality assurance steps in which citizen contributions supported validation and downstream analytical workflows.”

“A limited subset of studies (n=3, 4.9%) engaged citizens directly in data analysis^23,39,40^, including analytic interpretation, classroom-based data exercises, or community-supported modeling activities integrated into educational programs or surveillance evaluations.”

*Results* section 3.5.5 (added):

“In general, open-call recruitment was most common in app- and platform-based surveillance systems designed to maximize submission volume and geographic reach, whereas recruitment through community organizations, schools, and local leadership was more characteristic of place-based initiatives that depended on sustained engagement, training, or intervention activities.”

*Results* section 3.5.6 (added):

“Overall, citizen science initiatives commonly combined more than one function, with some platforms more strongly oriented toward quantitative surveillance and others placing greater emphasis on education, engagement, or local control while still contributing surveillance-relevant information.”

*Results* section 3.6.2 (added):

“In general, smartphone- and web-based platforms enabled scalable, geolocated reporting across wide areas, whereas sensor-based, trap-based, or educational tools were more often tied to specialized surveillance tasks, local monitoring, or training-oriented participation models.”

*Results* section 3.8 (added):

“Analytical approaches varied according to the type of citizen-generated data, the surveillance objective, and the extent to which studies addressed validation and uneven reporting effort.”

“Platforms based on low-burden public reporting, often supported by expert validation, were commonly used for species occurrence mapping, early detection, and spread tracking, particularly for invasive mosquitoes. Studies that produced more quantitative outputs, such as abundance estimates, hotspot mapping, temporal trend analysis, or risk modeling, more often incorporated stronger validation workflows, structured sampling, external benchmarking, or explicit effort-aware modeling. Community-based initiatives with deeper local engagement were more frequently associated with education, intervention, and locally grounded monitoring, even when they generated fewer analytically intensive outputs. These roles often overlapped, and several studies combined surveillance, engagement, and control objectives.”

*Discussion* section (revised):

“The review further suggests that the scientific value of citizen-generated mosquito data depends not only on the underlying validation workflow, but also on how participation model, effort-bias handling, and analytical objective are aligned. Low-burden public-reporting systems with strong expert validation were most often used for early detection, occurrence mapping, and spread tracking. In contrast, studies targeting quantitative outputs, such as hotspot mapping, temporal trend analysis, or risk modeling, more often required stronger validation, benchmarking, and effort-aware analytical approaches. Community-based initiatives with deeper local engagement more often oriented toward education, public engagement, and local vector-control activities. These roles were often overlapping rather than mutually exclusive. In this sense, citizen science can be understood as ground-truth, human-sensed, and socially mediated geospatial data that extend surveillance reach and timeliness while also reflecting the social geography of participation and associated reporting biases. This framing is particularly relevant during crises or operational disruptions, when routine field surveillance may be delayed or unable to scale and citizen-generated observations can provide rapid situational awareness^73^.”

******

**COMMENT**

In summary, the manuscript addresses an important and rapidly evolving area of research and provides a useful overview of existing citizen-science initiatives in mosquito-borne disease surveillance. However, the review would benefit from a deeper analysis of participation biases, validation procedures, and analytical approaches used to account for variations in participation effort. Expanding the discussion of governance and ethical transparency in relation to citizen participation, and developing a more integrated framework linking participation, validation, analysis, and outputs, would greatly enhance the contribution of the study. With these revisions, the manuscript could provide a more robust and informative synthesis of the current state of citizen-science approaches in mosquito surveillance.

**RESPONSE**

Many thanks for your thoughtful review. We agree and revised the manuscript substantially in response. Specifically, we strengthened the *Results* to better connect participation model, recruitment strategy, data type, validation workflow, effort-bias handling, and surveillance outputs, and to clarify the overlapping functional roles of different citizen science initiatives. We also expanded the *Discussion* on participation bias, validation rigor, effort-aware analysis, and ethical transparency, while adding a more integrated analytical perspective on how participation, validation, analysis, and outputs interact across citizen science systems. We believe your thoughtful review significantly strengthened the manuscript and improved its contribution as a synthesis of current citizen science approaches in mosquito surveillance.

******
